# Effect of Austerity Measures on Infant Mortality: Evidence From Greece

**DOI:** 10.1002/hec.70107

**Published:** 2026-04-16

**Authors:** Robert J. Kolesar, Rok Spruk

**Affiliations:** ^1^ Centre d’Etudes et Recherche sur le Développement International (CERDI), Université Clermont Auvergne Clermont‐Ferrand France; ^2^ University of Ljubljana Ljubljana Slovenia; ^3^ The University of Western Australia Perth Australia

**Keywords:** austerity, Greece, infant mortality, synthetic control method

## Abstract

Governments frequently adopt austerity policies when facing economic crises, yet their long‐term consequences for population health remain incompletely understood. This paper examines the impact of large‐scale fiscal austerity on infant mortality by exploiting the Troika‐led economic adjustment program implemented in Greece beginning in 2010 as a quasi‐experimental shock. Using the synthetic control method, we construct a counterfactual for Greece based on OECD and Union for the Mediterranean countries that did not experience austerity of comparable depth or duration. Relative to this counterfactual, Greece experienced a sharp and persistent increase in infant mortality following the onset of austerity. The divergence emerges immediately after 2010, remains statistically significant throughout the post‐intervention period, and shows little evidence of full reversion prior to the COVID‐19 pandemic. The estimated effect corresponds to an average 43 percent increase in the infant mortality rate. Mortality effects are larger for boys than for girls and are concentrated in the neonatal period. Accounting explicitly for the fertility decline, we estimate approximately 854 excess infant deaths cumulatively from 2010 to 2020. Extensive robustness checks support the findings. The results identify the total effect of austerity and highlight the importance of protecting early‐life health during fiscal consolidation.

## 1 Introduction

Achieving and maintaining equitable service access and vital public health outcomes necessitates large‐scale investments in health systems, capacity, and preventive care (Acemoglu and Johnson 2007). Large fiscal policy shocks to either government expenditure or taxation may have both short‐ and long‐term consequences for public health outcomes that affect equity, quality, and the provision of care (Corsetti 2012; Bailey and Goodman‐Bacon 2015; Fadlon and Nielsen 2021; Romer 2021). In 2009, Greece's infant mortality rate stood at around three deaths per 1000 live births, or roughly one half of the average mortality rate among OECD members. In the same year, the Eurozone's public debt crisis began in Greece (Chodorow‐Reich et al. 2023). To avoid bankruptcy, the country borrowed €256.6 billion from the International Monetary Fund (IMF), the European Union (EU), and the European Central Bank (Kentikelenis et al. 2011; Gourinchas et al. 2017). Greek public spending fell by 36% from 2009 to 2014 (Burki 2018, Commission, Economic, and Affairs 2023). The 2010 Troika Memorandum limited public expenditure on health to 6% of gross domestic product (Papademos et al. 2012), and by 2019 Greece was one of the few OECD member states with public health expenditure below 4% of GDP.

The austerity measures introduced under the Troika framework constituted a broad and tightly bundled policy package. They included severe reductions in government health expenditure, public employee salary cuts and layoffs, reductions in minimum wages, pensions, and welfare payments, and increases in taxes and special levies, among other measures. Together, these policies weakened the capacity of the state to address social risks such as unemployment, inequality, and poverty, while exacerbating pre‐existing structural weaknesses in the health‐care system (Kentikelenis and Papanicolas 2012; Adam and Papatheodorou 2016; Economou et al. 2015). Importantly, these changes plausibly affected infant health through multiple, potentially interacting channels, including health‐system capacity and access, household income and employment conditions, and broader macroeconomic stress.

In this paper, we exploit the introduction of the Economic Adjustment Program in Greece to examine the overall impact of fiscal austerity on infant mortality by estimating a counterfactual benchmark. Because the Greek adjustment program was implemented as a comprehensive fiscal and institutional package during a period of exceptional economic contraction, isolating any single mechanism, such as health‐care spending reductions, from other contemporaneous effects is not feasible with available aggregate data. Therefore, our estimand represents the total causal effect of the austerity episode as a whole on infant mortality, relative to a plausible counterfactual scenario in which such large‐scale austerity measures were not implemented.

We apply a quasi‐experimental synthetic control approach to address three voids in the literature. First, we use this method to quantify the effect of austerity measures on infant mortality in a setting where randomized or conventional difference‐in‐differences designs are not credible. Second, the analysis provides evidence on the longer‐term public health consequences of large fiscal policy shocks, rather than focusing solely on short‐run responses. Third, by disaggregating the effects by sex and extending the analysis to neonatal and post‐neonatal mortality, we provide additional evidence on sex‐related and age‐specific disparities in vulnerability to major macro‐fiscal interventions.

By comparing Greece with a stable donor pool of OECD and Union for the Mediterranean member states (excluding Syria and Lebanon due to instability and conflict) that did not undergo prolonged austerity programmes of comparable severity, we estimate the counterfactual scenario using the synthetic control estimator (Abadie 2021). A key advantage of the synthetic control method as a causal inference tool is that it flexibly accounts for both observed and unobserved confounders by matching the entire pre‐intervention outcome trajectory, rather than relying on the parallel‐trends assumption. For example, pre‐existing economic trends are implicitly controlled for through matching on the pre‐austerity infant mortality path, which reflects underlying economic and social conditions, including income dynamics (Barr et al. 2022). The method further allows unobserved confounders to vary over time by re‐weighting the counterfactual unit to closely reproduce the treated unit's pre‐intervention characteristics (Kreif et al. 2016; Barr et al. 2022). Against this backdrop, we document an excellent quality of fit between the infant mortality trajectories of Greece and its synthetic control group prior to the onset of austerity.

The results reveal that, relative to the synthetic counterfactual, the austerity episode in Greece is associated with a marked and persistent increase in infant mortality of approximately 43% in the post‐austerity period. The increase is around one third larger for boys and remains elevated for nearly a decade. These findings are robust to an extensive battery of sensitivity analyses and placebo tests. While the adjustment program formally ended in 2017 and infant mortality rates declined somewhat thereafter, the trajectory remained substantially above its counterfactual benchmark and did not fully revert to pre‐crisis trends by the end of the sample period.

Finally, we note an important interpretive consideration that is especially salient in the Greek context. Austerity coincided with a sharp decline in fertility, which may affect infant mortality rates mechanically through changes in the number of births and potentially through changes in the composition of births. Throughout the paper, our primary estimates are defined in terms of infant mortality rates. We therefore examine fertility dynamics in supplemental analyses and interpret mortality effects accordingly, distinguishing the overall impact of austerity on infant mortality from specific mechanisms operating through changes in risk conditional on birth versus changes in the composition of births.

The remainder of the article is organized as follows. Section 2 reviews the literature and policy background; Section 3 presents the identification strategy; Section 4 discusses the data and sample; Section 5 presents and discusses the results, robustness checks, and limitations; and Section 6 offers concluding remarks.

## 2 Background

### 2.1 Austerity

Austerity measures refer to policies that combine reductions in public expenditure with increases in taxation. Such measures can affect population health through multiple, potentially interacting pathways. A useful conceptual distinction in the literature separates direct effects on the health‐care system from indirect effects operating through broader socioeconomic conditions. Direct effects include cuts to healthcare services, reductions in coverage, and restrictions in access to care, often referred to as the health‐care effect (Stuckler et al. 2017). For example, increases in out‐of‐pocket payments can create access barriers and undermine financial risk protection (Grigorakis et al. 2018). In the Greek case, the World Health Organization reported that increases in healthcare user fees and co‐payments resulted in nearly one‐third of the poorest households incurring catastrophic medical expenditures by 2014 (Burki 2018).

Indirect effects of austerity operate through labor markets, household incomes, and social protection, and are commonly referred to as the social risk effect (Stuckler et al. 2017). Rising unemployment, income loss, and poverty are all associated with deteriorations in physical and mental health. For example, increases in unemployment are associated with higher probabilities of diabetes, infarction, ulcer, cirrhosis, and nervous disorders (Colombo et al. 2018). In Greece, adult unemployment rose from 6.6% in May 2008 to 16.6% in May 2011, while youth unemployment increased from 18.6 to 40.1% over the same period. At the same time, rising morbidity and reduced ability to afford private healthcare led to increased reliance on public health services (Kentikelenis et al. 2011). This surge in demand occurred alongside sharp reductions in public expenditure, placing additional strain on the system (Kentikelenis and Papanicolas 2012).

Beyond health outcomes, the three successive Economic Adjustment Programmes implemented in Greece are estimated to have had persistent negative effects on GDP per capita and long‐run growth (Efthimiadis et al. 2013). Sharp declines in disposable income combined with large increases in unemployment translated into a substantial rise in absolute poverty (Mitrakos 2014). Together, these developments underscore that the Greek austerity episode constituted a broad macro‐fiscal shock with likely impacts on health through multiple channels simultaneously.

### 2.2 Health System Effects

Prior to the 2009 economic crisis, the Greek health system faced significant structural challenges, including fragmented financing and governance, reliance on informal payments, inequities in access and quality of care, and inefficiencies such as oversupply of specialists alongside shortages of nurses (Mossialos et al. 2005, Davaki and Mossialos 2005, Charalambos Economou and Organization 2010, Economou et al. 2015, Commission, Economic, and Affairs 2023). These weaknesses shaped both the system's vulnerability to fiscal contraction and the way austerity measures translated into service delivery outcomes.

Health‐related components of the Greek adjustment programmes combined expenditure reductions with structural reforms aimed at improving efficiency and cost‐effectiveness. These included the creation of a national health insurance fund to pool resources, harmonization of contribution rates, centralized procurement of supplies, and cost‐containment measures such as reference pricing, expanded use of generics, electronic prescribing, and annual spending caps (Commission, Economic, and Affairs 2023). While these reforms were intended to address long‐standing inefficiencies, they were implemented during a period of acute fiscal retrenchment.

The immediate focus of health‐related austerity in Greece was on expenditure reduction, with uncertain short‐ and long‐term consequences for public health and health‐care delivery (Simou and Koutsogeorgou 2014). Large cuts to public health expenditure affected hospital staffing, operating budgets, and availability of essential supplies, exacerbating pre‐existing constraints (Ifanti et al. 2013). For example, health‐care staff salaries were reduced twice in 2010, maternal and child health services were cut by 73% between 2009 and 2012; and funding for public hospitals declined by roughly 50% between 2009 and 2015 (Burki 2018). These reductions coincided with rising hospital admissions as patients shifted from private providers and the number of uninsured individuals increased (Kentikelenis et al. 2011; Adam and Papatheodorou 2016). Consistent with this strain, mortality due to adverse events during medical treatment has been estimated at 242 excess deaths per month beginning in September 2008 (Laliotis et al. 2016).

The crisis further eroded social insurance revenues as unemployment rose, wages declined, and working hours were reduced (Burki 2018). Indicators of system performance reflect these pressures, including increased patient dissatisfaction and rising unmet need for healthcare (Zavras et al. 2016; Keramidou and Triantafyllopoulos 2018; Carr and Wolfe 2019; Filippidis et al. 2017). At the same time, it has been noted that the adjustment programmes also produced some positive institutional changes, such as standardization of benefits, restoration of universal coverage, pharmaceutical cost containment, and reforms to procurement and hospital payment systems (Economou et al. 2017). This mixed record highlights the complexity of evaluating health‐system performance during periods of deep fiscal consolidation.

### 2.3 Austerity, Income and Health Outcomes

A strong association between income inequality and infant mortality is well documented in the literature (Siddiqi et al. 2015). However, this relationship is not necessarily causal. Avendano (2012) argues that social policies that reduce infant mortality tend to cluster in countries with lower income inequality, even if their effects do not operate directly through income itself. This observation motivates closer examination of specific policy environments rather than reliance on cross‐sectional correlations.

At the micro and macro levels, infant mortality has been linked to both relative and absolute income as well as to unemployment (Dallolio et al. 2012). There is also growing evidence that stalled or reversed mortality improvements in several high‐income countries may be partly attributable to austerity policies following the global financial crisis (McCartney et al. 2022). Many European countries adopted fiscal consolidation measures after 2008, though with substantial heterogeneity in timing, severity, and institutional context (Staehr 2013).

**FIGURE 1:**
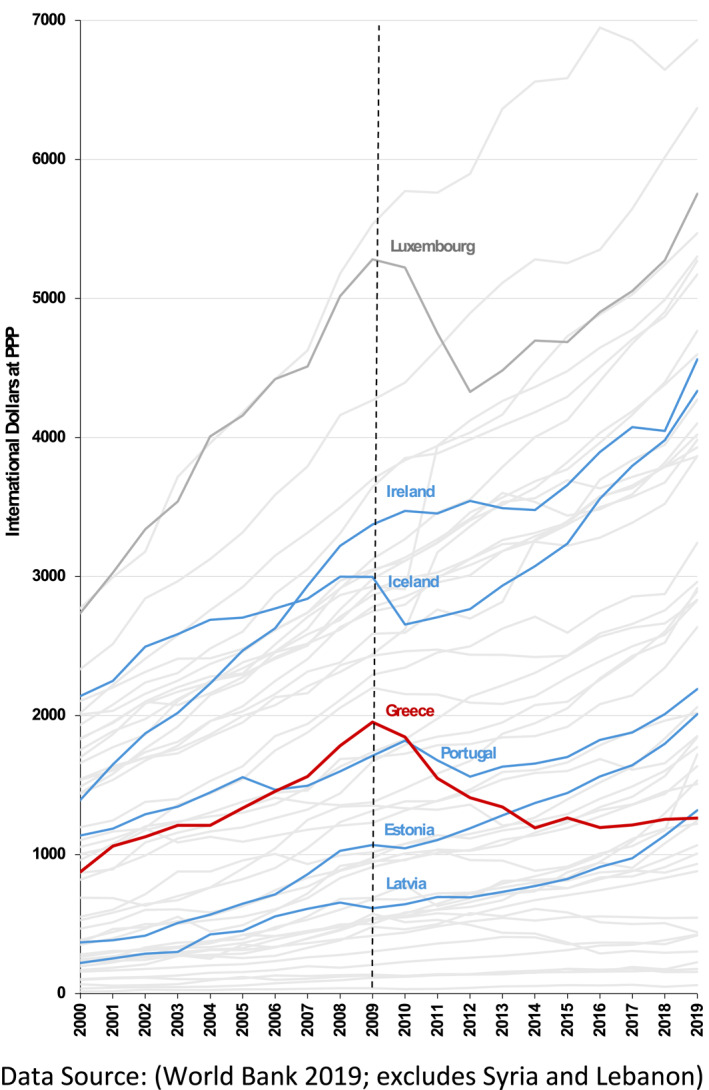
General government health expenditure per capita for OECD and Union for the Mediterranean member states from 2000 to 2019, in international dollars (at purchasing power parity). *Source:* (World Bank 2019; excludes Syria and Lebanon).

Figure 1 illustrates the evolution of per capita general government health expenditure for OECD and Union for the Mediterranean member states from 2000 to 2019. Comparative analyses of European responses to the crisis indicate that Greece, Spain, and Portugal implemented particularly strict austerity programmes, experienced deeper and longer economic contractions, and faced greater strain on their health‐care systems (Karanikolos et al. 2013). In contrast, Iceland rejected severe austerity measures by popular vote and experienced few discernible adverse health effects. Consistent with this pattern, Greece's general government health expenditure per capita declined by 39% between 2009 and 2014, from 1953 to 1191 international dollars. With the exception of Luxembourg, Greece is the only country in the donor pool to have implemented such dramatic and sustained reductions. By comparison, Iceland's health expenditure declined by 9.7% between 2009 and 2011 and began to recover thereafter.

A substantial body of evidence documents deterioration in self‐reported health, access to care, and service availability in Greece during the crisis, accompanied by increases in morbidity and mortality (Vandoros et al. 2013, K.N. Fountoulakis and Theodorakis 2014). Numerous studies have examined mortality outcomes during this period, including infant mortality, suicide, circulatory and infectious diseases (Kentikelenis et al. 2014a, 2014b; Laliotis et al. 2016; Filippidis et al. 2017; Kotzamanis et al. 2022; Zafeiris and Kostaki 2019; Zilidis and Hadjichristodoulou 2020; Siahanidou et al. 2019; Doetsch et al. 2023; Kubrin et al. 2022). A systematic review of 39 studies covering the period 2009–2013 concludes that the crisis was associated with deteriorations in population health, including increased mental health problems, suicides, epidemics, and worsening self‐rated health (Simou and Koutsogeorgou 2014).

### 2.4 Infant Mortality

Child mortality is heavily concentrated in the first year of life, with nearly half of all child deaths occurring among newborns (Quah 2016; World Health Organization 2022). The infant mortality rate, defined as deaths before the first birthday per 1000 live births, is widely used as a summary indicator of population health and social conditions (Centers for Disease Control 2024). This measure is particularly sensitive to structural factors such as living conditions, health‐system capacity, and socioeconomic development (Reidpath and Allotey 2003; Schell et al. 2007). In contrast, aggregate indicators such as life expectancy can mask important subgroup‐specific changes, including increases in suicide or cardiovascular mortality during periods of economic hardship (Granados and Rodriguez 2015; Fountoulakis et al. 2022).

The relationship between economic downturns and infant health is complex and context dependent (Bacigalupe et al. 2016). The literature reports heterogeneous findings, reflecting the coexistence of countervailing mechanisms (Stuckler et al. 2015). For example, evidence from the United States suggests that infants conceived during periods of high unemployment may exhibit lower rates of low birth weight, congenital malformations, and post‐neonatal mortality, potentially reflecting selection and behavioral responses among parents (Dehejia and Lleras‐Muney 2004). In Greece, nationwide evidence shows disparities in infant mortality trends by human development index and rural residence (Siahanidou et al. 2019). At the same time, multiple studies document adverse effects of economic crises and unemployment on infant mortality across countries (Alexander et al. 2011; Cruces et al. 2012; Zilidis and Hadjichristodoulou 2020). Catalano et al. (2011) describe this as a “net effect” framework, in which overall health outcomes reflect the balance of pro‐ and countercyclical forces operating across different population groups.

Adverse birth outcomes represent an important proximate risk factor. Pre‐term births and low birth weight are strongly associated with infant mortality, particularly when both conditions are present (Pusdekar et al. 2020). In Greece, the preterm birth rate increased by 16% between 2008 and 2010 (Vlachadis, Loukas, et al. 2024), alongside a 19% increase in low birth weight and a 23% rise in very low birth weight births (Vlachadis et al. 2025). Evidence also points to increases in low‐birth weight deliveries independent of maternal age and origin, as well as higher stillbirth rates among younger women during crisis periods (Zografaki et al. 2018). Cross‐country evidence further indicates that lower rates of low birth weight are associated with broader health‐system capacity, including coverage, hospital availability, and health‐care workforce density (Erasun et al. 2021).

At least 11 published studies examine infant mortality in Greece following the economic crisis. Seven document increases in infant mortality after the onset of the crisis (Filippidis et al. 2017; Kotzamanis et al. 2022; Doetsch et al. 2023; Zilidis and Hadjichristodoulou 2020; Siahanidou et al. 2019; Fountoulakis et al. 2022; Zafeiris and Kostaki 2019). Three find increases followed by partial recovery (Michas et al. 2014, K.N. Fountoulakis and Theodorakis 2014, Vlachadis, Loukas, et al. 2024). One study reports a temporary decline in infant mortality between 2008 and 2010 followed by a rebound (Rajmil et al. 2018), though the crisis onset in late 2009 complicates interpretation. Another study finds no evidence of a health crisis when comparing 2003–2007 with 2008–2012, again using periods that do not align closely with the timing of the crisis (Granados and Rodriguez 2015). Supporting Information S1: Appendix 1 summarizes data sources, methods, and findings across these studies.

### 2.5 Male Disadvantage

According to the United Nations Inter‐Agency Group for Child Mortality Estimation, child mortality is higher among boys than girls in all countries (You et al. 2015). A large body of literature documents excess male vulnerability in the perinatal and infant periods (Naeye et al. 1971; Ulizzi and Zonta 2002; Lawn et al. 2005; Sawyer 2012; Carlsen et al. 2013; Fottrell et al. 2015). Girls exhibit a biological survival advantage in early life, with lower susceptibility to birth complications, infections, and certain congenital conditions. As a result, the ratio of infant mortality among boys to girls exceeds one in settings where access to food and medical care is broadly equal (Sawyer 2012).

Environmental conditions before and during pregnancy may further amplify sex differences in infant outcomes. Preconception and prenatal stressors have been shown to disproportionately affect male fetal development (Pongou 2013). Consistent with this mechanism, a large study examining sex‐specific responses to macroeconomic conditions around birth finds that high unemployment rates are associated with a small but statistically significant reduction in birthweight among boys (Alessie et al. 2018). These patterns motivate the examination of sex‐disaggregated infant mortality responses in periods of severe economic stress.

## 3 Identification Strategy

### 3.1 Setup

This study examines the effect of the Greek austerity episode on the infant mortality rate by estimating the missing counterfactual trajectory in the absence of the post‐2009 adjustment program. To this end, we apply the synthetic control (SC) estimator (Abadie et al. 2010; Kreif and Diaz Ordaz 2019; Spruk and Kovac 2020; Bonander 2021; Gilchrist et al. 2023). The synthetic control approach constructs a weighted combination of comparison countries whose infant mortality dynamics closely reproduce Greece's pre‐austerity trajectory and uses this synthetic unit to approximate how infant mortality would have evolved had the austerity program not been implemented.

Because the Greek Economic Adjustment Program consisted of a bundled set of fiscal and institutional changes implemented during a period of severe macroeconomic contraction, our findings can be interpreted as the total causal effect of the austerity episode as a whole on infant mortality. This analysis does not isolate the contribution of any single policy component, such as reductions in health‐care spending, from other contemporaneous changes affecting households and the health system. Accordingly, the estimand throughout the paper is the overall difference between observed post‐2010 infant mortality in Greece and the counterfactual trajectory implied by the synthetic control.

Formally, we estimate the counterfactual infant mortality path of Greece by exploiting the implicit attributes of infant mortality dynamics in other OECD and Union for the Mediterranean member countries that share similar pre‐austerity characteristics but were not subject to austerity programmes of comparable severity and duration. This procedure yields a weighted combination of donor countries that best reproduces Greece's infant mortality trajectory prior to the onset of austerity. Details of the implementation are provided in Supporting Information S2: Appendix 2.

The central identifying assumption underlying the synthetic control approach is that the synthetic version of Greece's infant mortality trajectory in the pre‐austerity period provides a credible approximation of how mortality would have evolved in the absence of the austerity episode. Following the established literature, the most plausible way to achieve this is to match on the full pre‐intervention outcome path of infant mortality (Hinrichs 2012; Bilgel and Galle 2015; Kešeljević and Spruk 2024). When the outcome trajectory itself summarizes the relevant observed and unobserved determinants of mortality, auxiliary covariates become unnecessary and may even degrade performance (Kaul et al. 2015). Consistent with this logic, Kaul et al. (2022) show that optimization based on the full span of lagged outcomes more plausibly captures latent factors and can improve the performance of the synthetic control estimator while mitigating dimensionality concerns.

### 3.2 Inference

A well‐known limitation of the synthetic control method is that conventional large‐sample asymptotic inference is not available. To assess statistical significance, we therefore follow Abadie et al. (2010) and implement permutation‐based inference. Specifically, we conduct both in‐space and in‐time placebo tests to evaluate whether the estimated post‐austerity divergence in infant mortality for Greece is unusually large relative to placebo effects.

In the in‐space placebo analysis, the austerity episode is iteratively reassigned to donor‐pool countries that were not subject to deep, Troika‐imposed adjustment programmes, and synthetic controls are constructed for each placebo country. This generates a distribution of placebo treatment effects against which the magnitude of the Greek effect can be compared. In the in‐time placebo analysis, the austerity intervention is deliberately assigned to incorrect pre‐treatment years to test whether similarly large mortality divergences emerge in periods when no policy change occurred. The absence of comparable placebo effects strengthens the interpretation that the observed post‐intervention divergence reflects the impact of the austerity episode rather than spurious pre‐existing trends or coincidental shocks.

## 4 Data and Samples

### 4.1 Outcomes

The primary outcome of interest is the infant mortality rate. Using the estimates from United Nations Demographic Yearbook, we approximate the mortality rate as the total number of deaths in a given year of female or male children less than 1 year of age, divided by the total number of female or male live births in the same year, multiplied by 1000. The series is calculated from the estimates and projections of the number of child survivors at the age of one disaggregated by sex. Specifically, we compare the infant mortality trajectories between Greece and its donor pools both for boys and girls to better understand sex‐specific effect disparity of fiscal austerity.^1^

**FIGURE 2:**
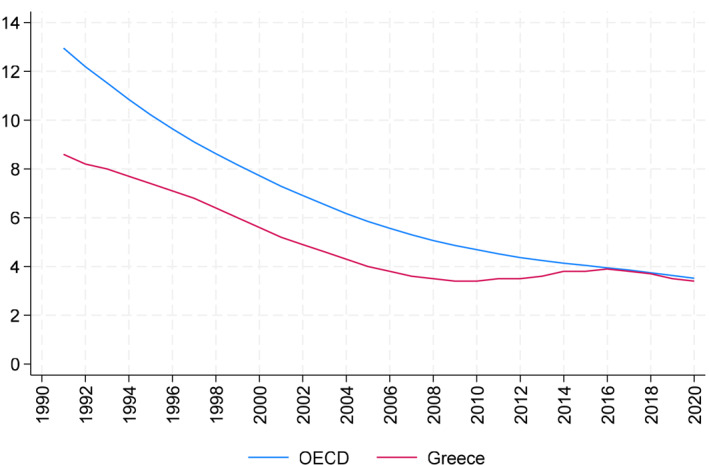
Infant mortality trajectories in OECD and Greece, 1991–2020.

Figure 2 depicts the overall infant mortality trajectories in Greece and OECD countries for the period 1991–2020. A broad comparison of trajectories shows that prior to the onset of the fiscal austerity measures, the Greek infant mortality trajectory appears to be substantially below the OECD average. Two detailed patterns emerge after the implementation of austerity measures. First, the austerity measures appear to have pushed the mortality trajectory of Greece closer to the OECD average and have decimated the country's noteworthy and substantial advantage compared to its OECD peers. Second, the infant mortality rate increased in absolute terms following the introduction of the austerity measures. Apart from Syria, Greece was the only nation in the Mediterranean basin and the only OECD member experiencing an absolute increase in infant mortality rate from 2009 until 2017. By the beginning of 2016, the Greek infant mortality trajectory evolved in tandem with the OECD average prior to the onset of the COVID‐19 pandemic. Post‐2017 infant mortality recovery may be attributed to the enactment of the National Health System law establishing two levels of primary healthcare: peripheral clinics and health centers. The later provide specialized services including maternal care and public health (Kampouraki et al. 2023). Consistent with the aggregate, cross‐country nature of the data, the analysis does not observe individual‐level maternal characteristics nor specific causal pathways, but rather focuses on estimating the total effect of the austerity episode on infant mortality.

**FIGURE 3:**
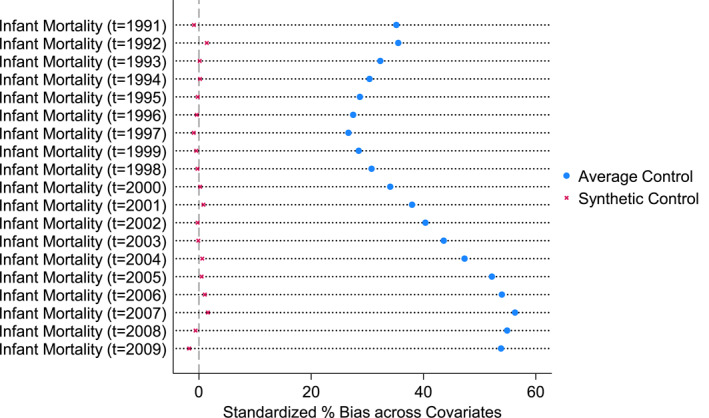
Standardized pre‐austerity outcome path imbalance.

Table 1 reports pre‐austerity infant mortality imbalance between Greece, its synthetic control group and the overall donor pool based on the full‐outcome path optimization of Greece and its convex hull of mortality attributes of the other donor pool members. The comparison of outcome balance show that in the absence of convex optimization and synthetic matching between Greece and the donor pool, a simple comparison of pre‐austerity mortality trajectories may not be able to uncover the overall mortality effect associated with the austerity measures. The overall bias between Greece and the donor pool in the absence of the convexity‐based analysis is between 26% and 56% higher than the observed mortality rate values for Greece. By contrast, the estimated bias between Greece and its synthetic control group in the pre‐austerity period is between 0.13% and 1.75%, respectively. Using full outcome path optimization to seek exact mortality attributes of donor pool countries that fall within the convex hull of Greek mortality dynamics nearly erases estimated bias from the comparison. It should be noted that low bias does not appear to be driven by disproportionately highly‐leverage pre‐austerity mortality rate in the benchmark years since the relative weight of each benchmark mortality rate is relatively evenly distributed across the pre‐austerity period, and, as such, does not pose an unresolved caveat for valid inference on the treatment effect of the austerity measures. Figure 3 graphically depicts standardized bias in the mortality rate comparison across the full span of benchmark years in the pre‐austerity period between Greece, its synthetic control group and the donor pool. The comparison indicates that the synthetic control group from the donor pool provides an excellent quality of the fit with the Greek infant mortality trajectory in the pre‐treatment period.

**TABLE 1:** Pre‐austerity outcome path imbalance.

	$\hat{V}$	$X_{t<T_{0}}^{Greece}$	Synthetic control group		Overall donor pool	
			$X_{t<T_{0}}^{0}$	Bias	Mean	Bias
${\text{Infant}\;\text{Mortality}}_{t=1991}$	0.061	8.60	8.52	−0.89%	11.62	35.16%
${\text{Infant}\;\text{Mortality}}_{t=1992}$	0.059	8.20	8.31	1.40%	11.11	35.49%
${\text{Infant}\;\text{Mortality}}_{t=1993}$	0.065	8.00	8.01	0.13%	10.58	32.30%
${\text{Infant}\;\text{Mortality}}_{t=1994}$	0.114	7.70	7.71	0.18%	10.04	30.38%
${\text{Infant}\;\text{Mortality}}_{t=1995}$	0.007	7.40	7.38	−0.22%	9.52	28.66%
${\text{Infant}\;\text{Mortality}}_{t=1996}$	0.004	7.10	7.07	−0.38%	9.05	27.46%
${\text{Infant}\;\text{Mortality}}_{t=1997}$	0.055	6.80	6.74	−0.94%	8.61	26.63%
${\text{Infant}\;\text{Mortality}}_{t=1998}$	0.104	6.40	6.37	−0.49%	8.22	28.45%
${\text{Infant}\;\text{Mortality}}_{t=1999}$	0.143	6.00	5.98	−0.27%	7.84	30.75%
${\text{Infant}\;\text{Mortality}}_{t=2000}$	0.051	5.60	5.61	0.21%	7.51	34.07%
${\text{Infant}\;\text{Mortality}}_{t=2001}$	0.065	5.20	5.24	0.80%	7.17	37.96%
${\text{Infant}\;\text{Mortality}}_{t=2002}$	0.027	4.90	4.89	−0.24%	6.88	40.33%
${\text{Infant}\;\text{Mortality}}_{t=2003}$	0.117	4.60	4.59	−0.11%	6.61	43.59%
${\text{Infant}\;\text{Mortality}}_{t=2004}$	0.045	4.30	4.33	0.59%	6.33	47.31%
${\text{Infant}\;\text{Mortality}}_{t=2005}$	0.017	4.00	4.02	0.48%	6.09	52.17%
${\text{Infant}\;\text{Mortality}}_{t=2006}$	0.001	3.80	3.84	1.04%	5.85	53.95%
${\text{Infant}\;\text{Mortality}}_{t=2007}$	0.039	3.60	3.66	1.59%	5.63	56.29%
${\text{Infant}\;\text{Mortality}}_{t=2008}$	0.003	3.50	3.48	−0.62%	5.42	54.89%
${\text{Infant}\;\text{Mortality}}_{t=2009}$	0.025	3.40	3.34	−1.75%	5.23	53.79%

*Note:* the table reports pre‐austerity infant mortality trajectory imbalance between Greece, its synthetic control group and the overall donor pool. The matrix $\hat{V}$ denotes the set of normalized variable weights in the diagonal and $X_{t<T_{0}}^{Greece}$ represents the mortality rates of Greece in each pre‐austerity period. The table also reports the corresponding bias between Greece and its synthetic control group, and Greece versus the overall donor pool.

**FIGURE 4:**
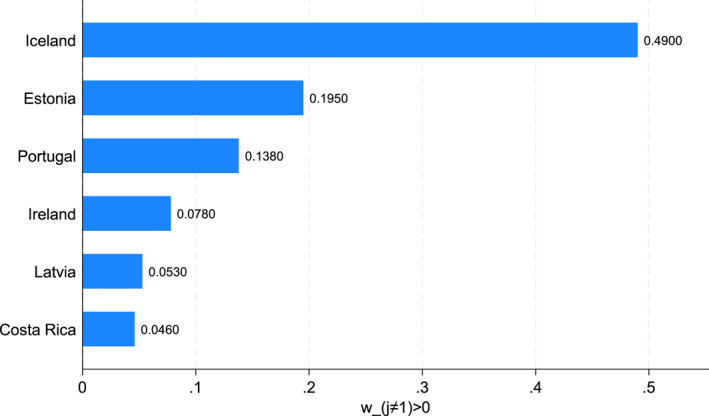
Composition of the synthetic control group for overall infant mortality.

Figure 4 shows the composition of the synthetic control group that best reproduces the pre‐austerity infant mortality trajectory of Greece but not subjected to the deep public health spending cuts. Therefore, the overall infant mortality of Greece in the pre‐austerity period is best synthesized by the implicit convex attributes of Iceland (49%), Estonia (19%), Portugal (13%), Ireland (7%), Latvia (5%), and Costa Rica (4%), respectively. The infant mortality trajectories of other donor pool countries fall outside the convex hull of Greek mortality characteristics and therefore do not receive a positive weight in the validation stage. Since none of the donor countries with positive weight had undergone such drastic reduction of public health expenditure in response to a foreign intervention by Troika nor underwent a deterioration of health outcomes in response to the economic crisis (Ásgeirsdóttir et al. 2014), it is unlikely that the donor pool is tainted by the confounding shocks that could be perceptible elsewhere and, thus, violate SUTVA (i.e., stable unit treatment value assignment) assumption. Although each individual donor country was affected by the economic downturn in the same period as Greece, the public health fiscal reforms and spending cuts in Greece have been rather unique in terms of magnitude, severity and duration as shown in Figure 1.

## 5 Results

### 5.1 Baseline

**FIGURE 5:**
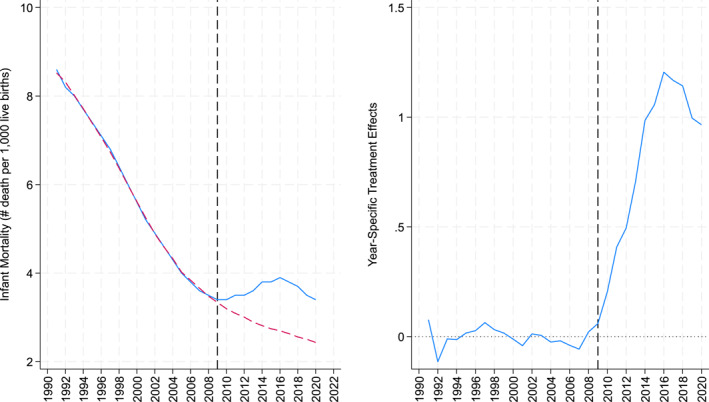
Infant mortality effect of austerity measures in Greece, 1991–2020.

We begin by estimating the total effect of the Greek austerity episode on infant mortality relative to a synthetic counterfactual. Figure 5 reports the infant mortality trajectories of Greece and its synthetic control group in the pre‐austerity period (1991–2009) and the post‐austerity period (2010–2020). The left panel of the figure shows an excellent pre‐intervention fit, with near‐zero discrepancy between Greece and its synthetic control prior to the onset of austerity. The right panel plots the year‐specific post‐intervention gaps, capturing deviations in infant mortality rates relative to the counterfactual trajectory.

Based on established benchmarks for synthetic control fit quality (Adhikari and Alm 2016), the root mean squared prediction error in the pre‐treatment period is very small (RMSE = 0.044), amounting to less than 1% of the pre‐treatment outcome level. This indicates a high‐quality reconstruction of Greece's pre‐austerity infant mortality path and supports the credibility of the counterfactual comparison. The estimated synthetic counterfactual implies that the austerity episode was followed by an immediate and persistent increase in the infant mortality rate. The divergence emerges sharply after 2009 and remains elevated throughout the post‐austerity period. Interpreted in terms of mortality rates, the estimated average post‐intervention gap corresponds to a substantial and sustained increase relative to the counterfactual scenario. The implied mortality excess is relatively small in the first year of austerity and increases steadily, peaking in the mid‐2010s before declining somewhat toward the end of the sample period prior to the COVID‐19 pandemic. Over the full 2010–2020 period, the cumulative mortality impact is large. Importantly, these calculations are based on observed births in each year and therefore reflect changes in the mortality rate conditional on realized fertility rather than assuming a fixed denominator.

**FIGURE 6:**
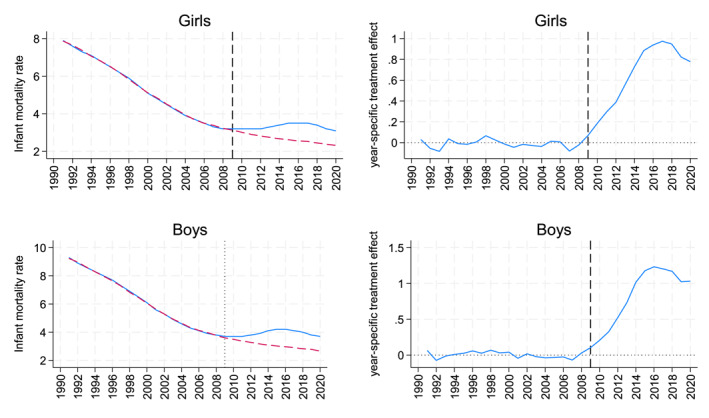
Sex‐disaggregated infant mortality effect of austerity measures in Greece, 1990–2020.

Figure 6 presents sex‐disaggregated synthetic control estimates. The increase in infant mortality is visibly concentrated among boys. On average, the post‐austerity mortality gap for boys is approximately 30% larger than that for girls, although the magnitude of the difference varies over time. In the first year of austerity, the mortality gap for boys exceeds that for girls by roughly 10%, by the peak of the divergence in 2016, the gap for boys is about 30% larger, and by the end of the pre‐pandemic sample period the disparity remains above 30%. A formal permutation‐based test rejects equality of the male and female effects at conventional levels, with the *p*‐value referring to the test of the *difference* between sex‐specific effects (*p*‐value< 0.001). These patterns are consistent with well‐documented male vulnerability in early life, which may be amplified under adverse economic and environmental conditions.

**FIGURE 7:**
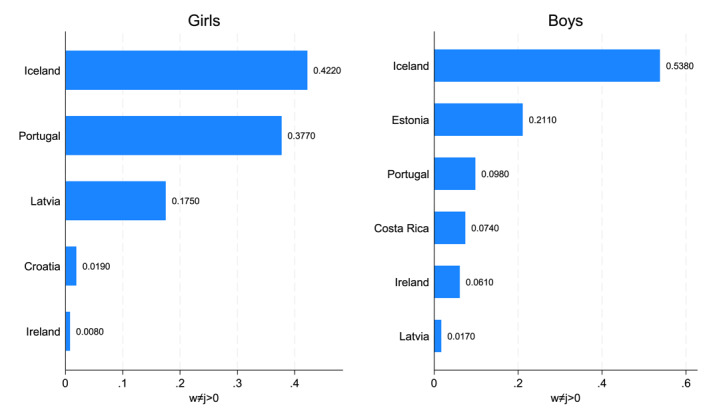
Sex‐disaggregated synthetic control groups.

Figure 7 reports the composition of the sex‐specific synthetic control groups. For girls, Greece's pre‐austerity infant mortality trajectory is best reproduced by a convex combination of Iceland (42%), Portugal (37%), Latvia (17%), Croatia (2%), and Ireland (less than 1%). For boys, the corresponding synthetic control places the largest weights on Iceland (54%), Estonia (21%), Portugal (9%), Costa Rica (7%), Ireland (6%), and Latvia (2%). The similarity between these sex‐specific donor compositions and the baseline synthetic control reported earlier confirms that the estimated mortality gaps are not driven by anomalous donor selection or unstable weights.

An important interpretive consideration arises from changes in fertility during the austerity period. A decline in births can mechanically affect infant mortality rates through the denominator and may also reflect changes in the composition of births. To assess this issue, we apply the synthetic control method to both the number of births and the birth rate in Greece over the same period. Relative to the synthetic counterfactual, the number of births declined by approximately 20% and the birth rate by about 11% following the onset of austerity, with both effects highly statistically significant (*p*‐value< 0.001). These findings are consistent with prior evidence that economic downturns are associated with delayed or foregone childbearing (Dehejia and Lleras‐Muney 2004).

Crucially, however, the aggregate data do not allow direct observation of changes in risk conditional on birth or in the socioeconomic composition of parents. We therefore refrain from attributing the observed increase in infant mortality to compositional changes per se. Instead, fertility decline is treated as an integral part of the austerity episode that may contribute mechanically to changes in mortality rates and potentially interact with underlying risk. To clarify the role of this channel, we report a bounding exercise in which we quantify the maximum increase in the infant mortality rate that could arise purely from a reduction in low‐risk births holding the number of infant deaths constant. This exercise shows that mechanical effects alone cannot account for the full magnitude and persistence of the estimated mortality divergence. Additional details on fertility dynamics and the bounding analysis are provided in Supporting Information S4: Appendix 4.

To aid interpretation, we translate mortality‐rate gaps into implied numbers of excess infant deaths. Because the synthetic control estimates are defined in terms of infant mortality rates (deaths per 1000 live births), we compute year‐specific excess deaths by multiplying the estimated rate gap by the observed number of live births in Greece in each year. This approach explicitly accounts for the large fertility decline documented in Supporting Information S4: Appendix 4. Applying this conversion yields approximately 852 excess infant deaths cumulatively over 2010–2020, or about 77 per year on average. Using sex‐specific births, the implied burden is larger for boys than for girls, approximately 451 versus 412 excess deaths over 2010–2020, consistent with the sex‐disaggregated mortality‐rate results. Full details, formulas, and year‐by‐year calculations are reported in Supporting Information S4: Appendix 4.

Table 2 summarizes the full post‐austerity distribution of overall and sex‐disaggregated infant mortality effects. Taken together, the baseline results indicate that the Greek austerity episode was followed by a large, persistent increase in infant mortality relative to a carefully constructed counterfactual, with effects that are systematically stronger for boys and robust to alternative specifications and inference procedures.

**TABLE 2:** Post‐austerity outcome balance and treatment effect composition.

	Overall			Girls			Boys		
	Actual infant mortality rate	Synthetic infant mortality rate	Treatment effect	Actual infant mortality rate	Synthetic infant mortality rate	Treatment effect	Actual infant mortality rate	Synthetic infant mortality rate	Treatment effect
2010	3.40	3.19	0.21	3.20	3.01	0.19	3.70	3.49	0.21
2011	3.50	3.09	0.41	3.20	2.91	0.29	3.70	3.37	0.33
2012	3.50	3.01	0.49	3.20	2.81	0.39	3.80	3.28	0.52
2013	3.60	2.89	0.71	3.30	2.74	0.56	3.90	3.17	0.73
2014	3.80	2.81	0.99	3.40	2.67	0.73	4.10	3.08	1.02
2015	3.80	2.74	1.06	3.50	2.62	0.88	4.20	3.02	1.18
2016	3.90	2.69	1.21	3.50	2.56	0.94	4.20	2.97	1.23
2017	3.80	2.63	1.17	3.50	2.52	0.98	4.10	2.90	1.20
2018	3.70	2.56	1.14	3.40	2.45	0.95	4.00	2.83	1.17
2019	3.50	2.50	1.00	3.20	2.38	0.82	3.80	2.78	1.02
2020	3.40	2.43	0.97	3.10	2.32	0.78	3.70	2.67	1.03
**Mean**	**3.63**	**2.79**	**0.85**	**3.32**	**2.64**	**0.68**	**3.93**	**3.05**	**0.88**

*Note:* This table reports post‐austerity infant mortality outcomes for Greece and its synthetic control for the period 2010–2020. Infant mortality rates are defined as the number of deaths before age one per 1000 live births. Actual refers to observed infant mortality in Greece, Synthetic refers to the counterfactual infant mortality rate estimated using the synthetic control method, and the treatment effect is the difference between the two (actual‐synthetic), expressed in deaths per 1000 live births. Columns report results for the overall population and separately by sex. Year‐specific effects are calculated as differences in mortality rates and therefore reflect changes in infant mortality conditional on realized births in each year. Summary statistics in the bottom row report simple averages across post‐austerity years. Tests of sex differences refer to the equality of male and female treatment effects; corresponding *p*‐values are reported in the text. The bold values at the bottom indicates average (mean) post‐treatment effect.

### 5.2 Robustness Checks

This section evaluates the robustness of the baseline findings and the credibility of inference. We focus on three complementary approaches: (i) in‐space placebo tests, (ii) in‐time placebo tests, and (iii) a broad set of additional sensitivity analyses reported in Supporting Information S3: Appendix 3.

#### 5.2.1 In‐Space Placebo Analysis

To assess whether the estimated post‐austerity divergence in infant mortality is statistically different, we conduct an in‐space placebo analysis by iteratively assigning the austerity intervention to each country in the donor pool and re‐estimating the synthetic control for each placebo‐treated unit. This procedure generates a reference distribution of post‐intervention mortality gaps under the null hypothesis of no treatment effect. The intuition is straightforward. If gaps of similar magnitude frequently arise for countries that were not exposed to deep austerity, then the estimated Greek effect could plausibly reflect chance variation or unobserved shocks unrelated to the intervention. Conversely, if Greece's post‐austerity gap is unusually large relative to the placebo distribution, conditional on comparable pre‐intervention fit, then the interpretation of a statistically meaningful effect becomes more credible. Following Abadie et al. (2010), inference proceeds in two steps. First, we compare the ratio of post‐to pre‐intervention root mean squared prediction errors (RMSEs). If the estimated mortality divergence is unique to Greece, this ratio should be markedly larger for Greece than for donor‐pool countries. Second, we compute quasi *p*‐values defined as the proportion of placebo units whose post‐/pre‐intervention RMSE ratio equals or exceeds that of Greece.

**FIGURE 8:**
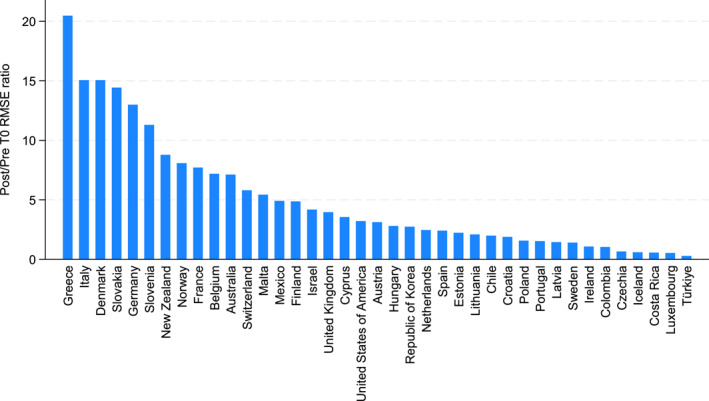
Comparison of post and pre‐austerity RMSE ratio.

**FIGURE 9:**
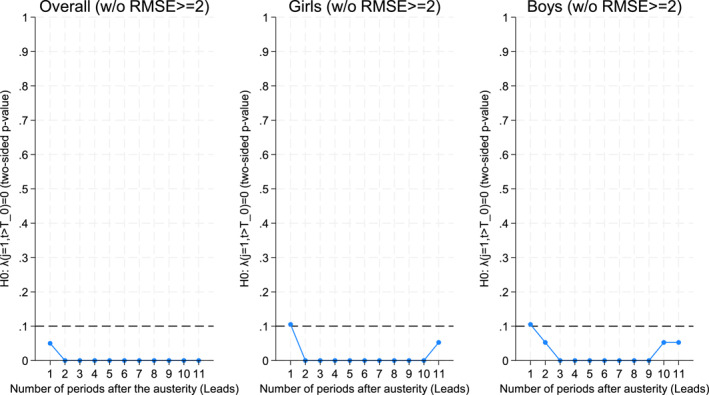
Inference on the infant mortality effect of austerity measures.

Figure 8 plots the distribution of post‐versus pre‐austerity RMSE ratios across donor‐pool countries. Greece exhibits the largest RMSE ratio in the sample, with no donor country approaching its magnitude. This result holds not only for the overall infant mortality specification, but also when the analysis is conducted separately for boys and girls. The absence of comparable placebo effects indicates that the estimated mortality divergence is highly distinctive to Greece. Figure 9 reports the intertemporal evolution of quasi *p*‐values over the post‐austerity period. The horizontal axis indexes years since the intervention, while the vertical axis reports the probability that a mortality gap of at least the observed magnitude arises by chance. Inference is based on the conventional threshold that *p*‐values below 0.10 indicate statistical significance.

A potential concern in placebo‐based inference is that donor‐pool units with very poor pre‐intervention fit may inflate the placebo distribution and obscure relative rarity. To address this, we restrict attention to placebo countries with pre‐austerity mean squared prediction errors no more than twice that of Greece. This conservative restriction focuses inference on units that, like Greece, are well matched prior to the intervention. Under this specification, quasi *p*‐values for the overall infant mortality effect fall below conventional significance thresholds immediately after the onset of austerity and remain near zero throughout the post‐intervention period. The null hypothesis that the observed mortality divergence occurred by chance can therefore be rejected consistently across years. Similar patterns are observed for the sex‐disaggregated specifications, confirming a statistically significant and persistent effect.

#### 5.2.2 In‐Time Placebo Analysis

**FIGURE 10:**
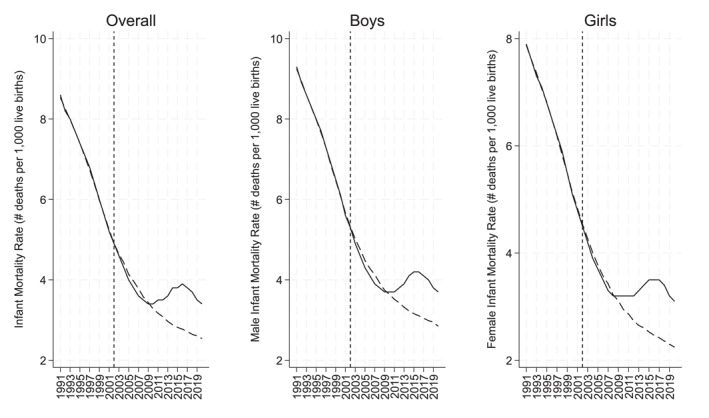
In‐time placebo analysis of the infant mortality effect of austerity measures in Greece, 1991–2020.

A separate concern for internal validity is that the estimated effect might reflect anticipation, pre‐existing reforms, or unrelated shocks occurring prior to the official start of the austerity program. If this were the case, assigning the intervention to an incorrect pre‐treatment year would be expected to generate similarly large post‐intervention divergences. To address this possibility, we conduct an in‐time placebo analysis by deliberately assigning the austerity intervention to an earlier year in the pre‐treatment period and re‐estimating the synthetic control. This falsification test evaluates whether the estimator produces large mortality effects when no true policy change occurred. Figure 10 reports the results of the in‐time placebo analysis for the overall and sex‐disaggregated infant mortality specifications. In all cases, the synthetic control reproduces Greece's mortality trajectory closely prior to the placebo intervention year. Crucially, no persistent divergence emerges following the false intervention date. Instead, mortality trajectories begin to diverge sharply only after the true onset of the austerity program in 2009. This pattern provides strong evidence against anticipation effects or spurious pre‐treatment shocks and reinforces the interpretation that the observed increase in infant mortality is associated with the austerity episode rather than unrelated temporal dynamics.

#### 5.2.3 Additional Robustness Checks

A comprehensive battery of additional robustness checks is reported in Supporting Information S3: Appendix 3. First, we apply a differential trend test (Spruk and Kovac 2020) and show that the post‐austerity period is characterized by a statistically significant upward structural break in the infant mortality trajectory that is distinct from pre‐austerity trends. Second, we perform a series of leave‐one‐out analyses (Abadie et al. 2015; Klößner et al. 2018) for the overall and sex‐disaggregated specifications. The estimated effects remain stable when influential donor countries, such as Iceland, are excluded sequentially, indicating that results are not driven by any single control unit. Third, exploiting sparsity conditions, we construct year‐specific 95% confidence bounds for the post‐intervention treatment effects. These bounds consistently exclude zero for most post‐austerity years, corroborating the baseline findings. Fourth, we vary the composition of the donor pool by restricting it to Euro‐Mediterranean OECD member states that are geographically and institutionally closer to Greece.^2^ The resulting estimates are quantitatively similar in magnitude and statistical significance to the baseline results for both boys and girls.

Fifth, we re‐estimate the effects using alternative counterfactual estimators. Applying the generalized synthetic control method (Xu 2017), which allows for interactive fixed effects, yields post‐austerity increases in infant mortality that closely match the baseline synthetic control estimates and survive in‐time placebo tests. Using the synthetic difference‐in‐differences estimator (Arkhangelsky et al. 2021), which relaxes both the parallel trends assumption and the convex hull requirement, similarly produces statistically significant increases in infant mortality. Finally, we implement a machine‐learning extension of the synthetic control method that permits extrapolation beyond the convex hull and allows both positive and negative weights (Hollingsworth and Wing 2020). This approach achieves excellent pre‐intervention fit and confirms a substantial post‐austerity increase in infant mortality. As an additional validation, we apply the synthetic control method to neonatal and post‐neonatal mortality series. The results indicate that approximately 92% of excess infant deaths occur in the neonatal period, with the remainder in the post‐neonatal stage, further reinforcing the interpretation that early‐life vulnerability is the primary driver of the observed mortality increase. Table 3 summarizes the estimated infant mortality effects across the different estimators and robustness specifications.

**TABLE 3:** Comparing the infant mortality effect of austerity measures across different variants of synthetic control estimator.

	Classical SCM	Generalized SCM	LASSO‐based SCM	Synthetic did
	(1)	(2)	(3)	(4)
Overall	+0.850*** (0.044)	+1.552*** (0.354)	+1.160*** (0.021)	+1.128** (0.375)
Boys	+0.850*** (0.042)	+1.338*** (0.343)	+0.967*** (0.012)	+1.186* (0.669)
Girls	+0.681*** (0.051)	+1.725*** (0.361)	+0.816*** (0.014)	+1.032** (0.338)

*Note:* the table reports the estimated effect of austerity measures on infant mortality rate in Greece for the period 1991–2020 using classical synthetic control method (Abadie et al. 2010, 2015), generalized synthetic control method (Xu 2017), LASSO‐based synthetic control method with countercyclical weights (Hollingsworth and Wing 2020), and synthetic difference‐in‐differences estimator (Arkhangelsky et al. 2021). Asterisks denote statistically significant average effect of austerity measures in 2010–2020 period at 10% (*), 5% (**), and 1% (***), respectively.

### 5.3 Limitations

This study has several limitations that are important for interpreting the results. First, the empirical design identifies the total effect of the austerity episode on infant mortality, rather than isolating the impact of specific policy instruments or causal pathways. The Greek austerity programmes included large and abrupt reductions in public health expenditure, but they also coincided with substantial changes in household income, employment, poverty, and social protection. Data limitations prevent us from separately identifying the contribution of individual components, such as cuts to healthcare staffing, hospital budgets, or preventive services, relative to broader socio‐economic shocks. Accordingly, the estimated effects should not be interpreted as attributing the observed increase in infant mortality exclusively to healthcare spending reductions. Instead, the results capture the combined impact of the austerity episode. We therefore rely on the existing literature to contextualize plausible channels and to distinguish between direct health‐system effects and indirect social‐risk effects.

Second, related to this point, the analysis does not identify disease‐specific causes of infant mortality or intermediate maternal health outcomes, nor does it directly observe changes in healthcare infrastructure, service quality, or utilization at a sub‐national or facility level. While prior work documents sharp contractions in maternal and child health services during the Greek austerity period, the aggregate data available for consistent cross‐country comparison do not permit a disaggregated assessment of morbidity pathways. Future research using detailed administrative, clinical, or survey‐based data would be needed to disentangle these mechanisms.

Third, infant mortality is measured as a rate per 1000 live births, which raises interpretive issues when fertility changes substantially. As documented in Supporting Information S4: Appendix 4, Greece experienced a pronounced decline in births following the onset of austerity. This decline affects the denominator of the mortality rate and may also reflect changes in the composition of births. To address this concern, we explicitly translate mortality‐rate gaps into implied numbers of excess infant deaths using observed, and sex‐specific live births, and we interpret the results as conditional on realized fertility. While this approach transparently incorporates the fertility decline, the available data do not allow us to distinguish between increased mortality risk conditional on birth and compositional changes in who gives birth. The findings should therefore be interpreted as reflecting the overall population‐level mortality burden associated with the austerity episode, rather than changes in individual‐level risk alone.

Fourth, as with all applications of the synthetic control method, inference relies on the quality of the pre‐intervention fit and on placebo‐based procedures rather than large‐sample asymptotics. We address this limitation through extensive in‐space and in‐time placebo tests, conservative restrictions on pre‐intervention fit, and a wide range of alternative estimators reported in Supporting Information S3: Appendix 3. While these exercises consistently support the robustness of the results, the interpretation remains comparative and counterfactual in nature.

Fifth, data availability precludes a sub‐national analysis. Infant mortality, fertility, and key predictors are not consistently available at comparable sub‐national levels across countries in the donor pool. As a result, we cannot assess within‐country heterogeneity in exposure to austerity measures or exploit regional variation in impacts. Sub‐national analyses using harmonized within‐country data would be a valuable extension for future research.

Finally, broader data‐quality concerns merit consideration. The Greek economic crisis was preceded in part by deficiencies in fiscal reporting (Rauch et al. 2011), and it is unlikely that routine administrative statistics were entirely immune to the effects of severe budget cuts. That said, mortality rates and mortality‐based indicators are generally regarded as among the most reliable measures of population health (Granados and Rodriguez 2015). The strong pre‐intervention fit, the consistency of the mortality series across sources, and the uniqueness of the post‐intervention divergence relative to donor countries all mitigate concerns that the results are driven by reporting artifacts. Taken together, these limitations emphasize that the paper provides evidence on the aggregate and persistent mortality consequences of a large‐scale austerity episode, rather than on narrowly defined policy levers or biological mechanisms. Within these bounds, the findings remain robust and informative for understanding the public‐health implications of sharp fiscal contractions.

## 6 Discussion and Conclusion

This study examines the relationship between large‐scale fiscal austerity and infant mortality by exploiting the Troika‐led economic adjustment program implemented in Greece beginning in 2010 as a quasi‐experimental shock. Using the synthetic control method, we construct a transparent counterfactual for Greece based on a stable donor pool of OECD and Union for the Mediterranean countries that did not experience austerity of comparable depth or duration. The resulting synthetic control reproduces Greece's pre‐austerity infant mortality trajectory with excellent precision, providing a credible benchmark against which to assess post‐intervention divergence.

Relative to this counterfactual, Greece experienced a sharp and persistent increase in infant mortality following the onset of austerity. The divergence emerges immediately after 2010, remains statistically significant throughout the post‐austerity period, and shows little evidence of full reversion prior to the COVID‐19 pandemic. The magnitude of the estimated effect implies a substantial and enduring mortality burden among infants, the most vulnerable segment of the population. Importantly, and consistent with the limitations discussed above, these estimates capture the total effect of the austerity episode rather than the impact of any single policy instrument. While deep reductions in public health expenditure were a central feature of the adjustment program, austerity also operated through income losses, unemployment, poverty, and weakened social protection. The results should therefore be interpreted as reflecting the combined consequences of a broad fiscal contraction rather than a narrow healthcare‐only channel.

The increase in infant mortality is not only large but heterogeneous. We document systematically larger effects for boys than for girls, with mortality‐rate gaps approximately one third higher among boys throughout much of the post‐austerity period. This pattern aligns with a well‐established literature documenting a biological and environmental survival disadvantage among male infants, particularly under conditions of heightened stress and resource scarcity. The concentration of excess deaths in the neonatal and early infancy period, approximately 92% of the total, further bolster the sensitivity of early‐life outcomes to adverse macroeconomic and institutional shocks.

The credibility of these findings is reinforced by an extensive set of robustness checks. In‐space placebo tests show that Greece's post‐austerity mortality divergence is unique within the donor pool and not replicated by countries with comparable pre‐intervention fit. In‐time placebo analyses confirm that assigning the intervention to incorrect pre‐treatment years does not generate spurious effects, ruling out anticipation or unrelated pre‐existing trends. The results are robust to alternative donor pools, leave‐one‐out exercises, and a wide range of alternative estimators, including generalized synthetic control, synthetic difference‐in‐differences, and machine‐learning extensions of the synthetic control method. Together, these exercises support a causal interpretation of the post‐austerity mortality divergence as a consequence of the fiscal adjustment episode.

Our findings are consistent with, and extend, a growing literature on the health consequences of austerity. Prior work has shown that high austerity is associated with increased material deprivation, child poverty, and low birth weight (Rajmil et al. 2020), and that IMF programmes are linked to weakened health systems and impeded progress on child and maternal health (Stuckler et al. 2017). Other studies document adverse effects of austerity on suicide, adult mortality, and life expectancy in Europe (Karanikolos et al. 2013; Kubrin et al. 2022; McCartney et al. 2022). Our contribution is to provide counterfactual‐based, long‐horizon evidence on infant mortality, allowing the mortality toll of austerity to be quantified relative to a credible benchmark.

Relative to earlier studies of infant mortality in Greece, which largely focused on short‐term correlations or descriptive trends, our analysis demonstrates that the health consequences of austerity are not merely transitory. As emphasized by Kotzamanis et al. (2022) and Bacigalupe et al. (2016), population health deterioration may unfold gradually and persist long after the initial shock. Consistent with this view, our results suggest that Greece's infant mortality trajectory was durably derailed during the austerity period, with effects that extend well beyond the immediate years of fiscal contraction.

The policy implications are clear. Fiscal consolidation during economic downturns entails trade‐offs that extend beyond macroeconomic balances and sovereign risk. When adjustment programmes involve sharp and prolonged contractions in public spending and social protection, the costs may include lasting damage to public health—costs that are borne, in part, by infants and children and that cannot be easily reversed. While governments must safeguard fiscal sustainability, our findings underscore the importance of protecting core public investments that sustain early‐life health. Preserving access to healthcare, maternal services, and social support during downturns is not only a matter of equity but also of long‐term economic and human development. Policies that neglect these dimensions risk trading short‐term fiscal relief for enduring social, demographic, and economic losses.

## Conflicts of Interest

The authors declare no conflicts of interest.

## Supporting information


Supporting Information S1

Supporting Information S2

Supporting Information S3

Supporting Information S4

## Data Availability

The data that support the findings of this study are available from the corresponding author upon reasonable request.

## Endnotes

^1^
Because infant mortality is defined as deaths per 1000 live births, changes in fertility affect the denominator of the outcome measure; we therefore examine fertility and births explicitly in the supplemental analyses and interpret mortality‐rate effects accordingly.

^2^
Croatia, Cyprus, France, Israel, Italy, Malta, Portugal, Slovenia, Spain and Türkiye.
