# Supplementary material for: Effect of Austerity Measures on Infant Mortality: Evidence From Greece
Source: Health Econ. 2026 Apr 16;35(8):1175–91. doi: 10.1002/hec.70107 (PMC13327493; doi:10.1002/hec.70107)
Supplement: Supplementary file 1 — Supporting Information S1 [file HEC-35-1175-s001.docx]

**Supplementary Appendix 1: Greek Mortality Studies**

| **Study** | **Data** | **Methods** | **Findings** |
| --- | --- | --- | --- |
| ***Increased*** ***Mortality*** |  |  |  |
| K. N. Zafeiris and A. Kostaki, "Recent mortality trends in Greece" 2019. | Greek Statistical Authority | Analysis of life expectancy at birth and several other ages by smoothing of morbidity probabilities (using Heligman-Pollard with cubic splines) to life tables by sex. | Appearance of the first effects of the economic crisis on mortality levels at the ages of 0 and 15 years, given that a significant time lag is needed for the effects of the economic and social crisis which afflicted in Greece to materialize. |
| F. T. Filippidis, V. Gerovasili, C. Millett, and Y. Tountas, "Medium-term impact of the economic crisis on mortality, health-related behaviours and access to healthcare in Greece", 2017. | Hellas Health surveys (national household data) | Interrupted time series analysis to compare trends in standardized mortality by cause before and during the crisis. | Standardized mortality rates for suicides and **infant mortality significantly increased during the crisis compared to pre-existing trends**; mortality from respiratory diseases and transport accidents decreased; the prevalence of unmet need for healthcare significantly increased (RR = 2.10) as did the proportion of people paying out-of-pocket for healthcare (RR = 1.69) between 2010 and 2015 |
| B. Kotzamanis, K. Zafeiris, and A. Kostaki, "Mortality in Greece before and during the recent economic recession: Short-terms effects of the economic austerity", 2022. | Hellenic Statistical Authority | Mortality trends in Greece from 1980 to 2019 with comparison of before and after 2011. | Slowdown in the growth of life expectancies after 2010; **increase in infant mortality** and death probability from suicide and certain diseases of the circulatory and respiratory system, infectious and parasitic diseases; large drop in road accident deaths |
| J. N. Doetsch et al., "2008 economic crisis impact on perinatal and infant mortality in Southern European countries" 2023. | Eurostat, the Organisation for Economic Cooperation and Development (OECD) and Statistics Portugal | Generalized Linear Regression/Poisson joinpoint regression model to explore mortality trend changes between 2000 and 2018; country disparities were analyzed using mixed-effects multilevel models. | Decreasing pace of infant mortality was attenuated after 2009 in Spain, Italy and Portugal; **in Greece a positive trend (i.e. increase) was found after the 2008 crisis**. IMR and PMR were significantly associated with socioeconomic indicators in all four countries |

| **Study** | **Data** | **Methods** | **Findings** |
| --- | --- | --- | --- |
| C. Zilidis and C. Hadjichristodoulou, "Economic crisis impact and social determinants of perinatal outcomes and infant mortality in Greece" 2020. | National vital statistics of Greece  Six mortality metrics, including infant mortality. | Average Annual Percent Changes were calculated by the period before and after 2008. The expected rates of 2009–2016 and the observed-to-expected rate ratios were calculated. Correlation and multiple linear regression analyses were used to test the impact of socio-economic variables on health outcomes | **Reversal of downward trends** of post-neonatal, infant, and neonatal mortality is observed since 2009. **All observed values of 2009–2016 were found significantly higher than the expected by 12–34%.**  Economic crisis was associated with remarkable adverse effects on perinatal outcomes and infant mortality, **mainly determined by long-term unemployment and income reduction** |
| T. Siahanidou et al., "Disparities of infant and neonatal mortality trends in Greece during the years of economic crisis by ethnicity, place of residence and human development index: a nationwide population study" 2019. | Hellenic Statistical Authority | Poisson, joinpoint regression and interrupted time series (ITS) analyses during the period 2004–2016 | **Increasing, but non-significant trends of infant and early neonatal mortality** (i.e. <7 days) among Greek mothers’ infants following the crisis;  ITS analyses found significant trend increases in IMR, late neo-natal mortality (7-27 days) and post-neonatal mortality (28-364 days) after 2012, and in early neonatal mortality (<7 days) after 2010. This study also explored the role of socio-demographic factors in the years of the crisis. |
| K. Fountoulakis, N. Fountoulakis, P. Theodorakis, and K. Souliotis, "Overall mortality trends in Greece during the first period of austerity and the economic crisis (2009-2015)" 2022. | World Bank, the Organisation for Economic Co-operation and Development, Eurostat, and the Hellenic Statistics Authority | Separate linear regression models were developed for the periods before and after the crisis and were compared. | **Total infant mortality rates show an overall rising trend since 2009**, however the interpretation is unclear because of changes in the socio-economic composition of couples giving birth (e.g. a reduction in the absolute number of deliveries among wealthier couples).  Note: Authors’ rationale about socio-economic composition is not supported by evidence in the study and does not seem to acknowledge that the economic crisis increased poverty (see Zilidis and Hadjichristodoulou, Siahanidou et al.). |

| **Study** | **Data** | **Methods** | **Findings** |
| --- | --- | --- | --- |
| ***Increased infant mortality with recovery*** | | |  |
| Vlachadis N, et al. Infant, Neonatal, and Post-neonatal Mortality in Greece: A Nationwide Time-Trend Analysis. Cureus. 2024 | Hellenic Statistical Authority | Mortality rate time trends were evaluated using joinpoint regression analysis, and the annual percent changes (APC) and the overall average annual percent change were calculated. | In 2008, IMR reached its all-time low of 2.7 per 1,000 live births, down 16.6-fold from its peak at 44.1 per 1,000 live births in 1957**. This improving trend was reversed following the onset of the economic crisis in the country, leading to a 57% increase in IMR from 2008 to 2016, with an upward trend APC of 3.4.** In the period 2016-2022, APC improved by −3.7. |
| G. Michas, A. Varytimiadi, I. Chasiotis, and R. Micha, "Maternal and child mortality in Greece" 2014 | Hellenic Statistics Authority | Comparison of infant and child mortality rates from 2003-2012 | In the first 2 years of the crisis (2009–10), infant mortality rate and child mortality rate gradually increased, and after 2010 it started to decline again |
| K. N. Fountoulakis and P. N. Theodorakis, "Austerity and health in Greece," 2014. | Hellenic Statistics Authority | Not stated | Infant mortality unstable trends with a decrease in 2008, followed by an equivalent increase in 2009 and 2010, then returning to lower levels. |
| ***Decreased mortality*** | | |  |
| L. Rajmil, D. Taylor-Robinson, G. Gunnlaugsson, A. Hjern, and N. Spencer, "Trends in social determinants of child health and perinatal outcomes in European countries 2005–2015 by level of austerity imposed by governments: a repeat cross-sectional analysis of routinely available data" 2018. | International Monetary Fund, the Organization for Economic Co-operation and Development and Eurostat | Longitudinal ecological study of country-level time trends in perinatal outcomes and SDCH and from 2005 to 2015. | In Greece, infant mortality from 2005-2007 was 3.66%; 2008-2010 3.2% and 2012-2015 3.60%; Overall, the study found that countries that implemented more severe austerity measures have experienced increasing low birth weight and for families with primary education also increasing material deprivation, worsening the negative impact of economic crisis. |
| ***No excess mortality*** | | |  |
| J. A. T. Granados and J. M. Rodriguez, "Health, economic crisis, and austerity: A comparison of Greece, Finland and Iceland" 2015. | World Health Organization, OECD statistics, and European Commission Eurostat data | Comparison of the linear trend slopes of population health and health services metrics from 2003-2007 with 2008-2012 in Greece, Finland and Iceland. | Evidence does not support the claim that there is a health crisis in Greece; the three countries most indicators of population health continued improving after the Great Recession started. |
