# Supplementary material for: Effect of Austerity Measures on Infant Mortality: Evidence From Greece
Source: Health Econ. 2026 Apr 16;35(8):1175–91. doi: 10.1002/hec.70107 (PMC13327493; doi:10.1002/hec.70107)
Supplement: Supplementary file 2 — Supporting Information S2 [file HEC-35-1175-s002.docx]

**Supplementary Appendix 2: Synthetic control method and the identification strategy**

Previous studies use a variety of methods including life expectancy analysis, interrupted time series, time trends, and pre-post comparisons. We apply Synthetic Control Method (SCM) which relaxes the parallel trend assumption to build a more flexible latent factor model where the counterfactual is projected from the extant mortality attributes of Greece before austerity measures. Moreover, SCM enables a direct estimate of excess mortality attributable to the study event.

This study evaluates the effect of austerity policies by estimating the missing counterfactual realization of the mortality dynamics and simulating its trajectory in the hypothetical absence of the austerity policy and designated cuts in public healthcare. We apply the synthetic control estimator (Abadie et. al. 2010; Kreif and Diaz-Ordaz 2019; Bonander 2021) to estimate the effect of austerity on infant mortality rate in Greece. Through the application of the potential outcomes framework (Rubin 1973), the infant mortality trajectories of Greece can be simulated through the combination of attributes of other countries that have similar characteristics but have not undergone a severe austerity during the study period. This approach allows the construction of a counterfactual representation of Greece by making use of weighted averages reflecting the resemblances of infant mortality characteristics before the introduction of austerity policies.

It should be noted that the impact of austerity policies is reflected by the infant mortality difference between Greece and the estimated counterfactual absent deep public health spending cuts in the post-intervention period. The synthetic control group that best reproduces infant mortality attributes of Greece prior to the austerity policies allows us to predict and infer the plausible level of mortality rate if the austerity cuts in public health spending were hypothetically never implemented. Provided that the synthetic control group is not tainted by the presence of similarly harsh spending reductions, the outcome difference plausibly reflects the effect of austerity policies on infant mortality rate.

*S2.1 Setup*

Suppose that we observe $J+1$ countries over $t=1,2,\ldots T$ period where $J$ denotes the country affected by a deep reduction in public health spending and $\left\{ 2,\ldots J+1 \right\}$ represent the donor pool of OECD countries not affected by the intervention. As a mimic of the treatment, austerity policy occurs at time $T_{0}$ and lasts in the post-intervention period so that $t<T_{0}<T.$Our aim is to estimate the impact of the health-related austerity on infant mortality as the underlying outcome variable. Without the loss of generality, let $Y_{i,t}^{N}$ denote the outcome for country $i$ at time $t$ in the absence of the austerity health policy. By contrast, let $Y_{i,t}^{I}$ be the outcome for country $i$ that would be observed if it were exposed to the austerity. By assuming that the austerity has no effect on the outcome of interest in the pre-intervention period, it follows that $Y_{i,t}^{N}=Y_{i,t}^{I}$ for all $i$ and $t<T_{0}+1$ and we further assume that a harsh form of austerity in health care only affects Greece. For each $t>T_{0}$, the effect of the austerity on infant mortality can be written as follows:

$\theta_{1,t}=Y_{1,t}^{I}-Y_{1,t}^{N}=\underset{\mathrm{observed}}{\underbrace{Y_{1,t}}}-\underset{\mathrm{counterfactual}}{\underbrace{Y_{1,t}^{N}}}$ (1)

where the key challenge is to construct the unobserved counterfactual and estimate $\theta$ accordingly. To reconstruct the missing counterfactual scenario, we rely on the latent factor model that accommodates pre-$T_{0}$ infant mortality path to estimate the unobserved counterfactual component:

$Y_{i,t}^{N}=\eta_{t}+\pi_{t}\cdot Z_{i}+\mu_{t}\cdot\phi_{i}+\varepsilon_{i,t}$ (2)

where $\eta_{t}$ is an unobserved common factor that mimics time-fixed effects, $Z_{i}\in\mathbb{R}^{r}$ is a vector of observed time-varying and time-invariant covariates unaffected by the economic crisis, $\pi_{t}^{'}\in\mathbb{R}^{r}$ is a vector of known parametric factor loadings, and $\mu_{t}^{'}\in\mathbb{R}^{F}$ is a vector of common unobserved factors, and $\phi_{t}^{'}\in\mathbb{R}^{F}$ is a vector of unknown factor loadings. The term $\varepsilon_{i,t}$ denotes transitory outcome shocks with $\varepsilon_{i,t}\mathbb{\sim N}\left( 0,1 \right)$ form of distribution. The key advantage of the latent factor model is to allow heterogeneous response of outcomes to multiple unobserved factors ($\mu_{t}\cdot\phi_{i}$) and embeds time trend models therein. The proposed latent factor model implicitly assumes that the number of common unobserved factors ($\mu_{t}$) is fixed over time which invokes the absence of structural breaks. The basic intuition behind the latent factor model is to reweigh the control group so that the synthetic version of Greece will match its observed $Z_{i}$. Hence, $\phi_{i}$ will be matched by default and the set of unobserved common factors will not be projected out of the outcome generating process allowing for heterogeneous response to multiple unobserved factors. Consider a simple $J\times1$ single dimensional vector of weights $W=\left( w_{2},\ldots,w_{J+1} \right)'$ where $w_{j}\geq0$ for $j=2,\ldots,J+1$ and $\sum_{j=2}^{J+1} w_{j}=1$. Notice that each particular value of $W$ represents the weighted average of the implicit mortality attributes of the countries from a donor pool where $w_{j}\neq0$. Let $\mathbf{X}$ represent pre-$T_{0}$ mortality trajectory combined into a single vector that is partitioned into $\mathbf{X}_{Greece}$ and $\mathbf{X}_{0}$ where the former captures the values of matching variables for Greece and $\mathbf{X}_{0}$ represents the values of the variables for the countries unaffected by the austerity. To appropriately measure the discrepancy between Greece and the unaffected countries, we estimate $W$ through a nested optimization method using constrained quadratic programming routine to find best-witting set of weights conditional on the matrix of covariates. It is based on the algorithm using the Vanderbei (1999) interior point method to solve the constrained quadratic programming problem, and is implemented via C++ plugin with 5 percent margin for the constraint violation tolerance using high-speed maximum likelihood approach as our default. We use a standard inner optimization to minimize a simple Euclidean distance between $\mathbf{X}_{Greece}$ and $\mathbf{X}_{0}$ to find the best-fitting weight set:

$\boldsymbol{W}^{*}=\underset{W}{\mathrm{argmin}} \left\| \mathbf{X}_{Greece}- \mathbf{X}_{0}W \right\|_{\boldsymbol{V}}=\sqrt{\left( \mathbf{X}_{Greece}- \mathbf{X}_{0}W \right)'\mathbf{V}\left( \mathbf{X}_{Greece}- \mathbf{X}_{0}W \right)}$ (3)

where $\boldsymbol{V}$ is a symmetric diagonal matrix with positive components wherein $k$ diagonal elements $\left( v_{1},\ldots.v_{k} \right)$ represent the predictive weights of the pre-$T_{0}$ fitted variables. In the next step, in the outer optimization, $\boldsymbol{V}$ can be estimated to minimize mean square prediction error in the pre-austerity period such that:

$\boldsymbol{V}^{*}=\underset{V}{\mathrm{argmin}} \left( \mathbf{Y}_{Greece}-\mathbf{Y}_{0}\mathbf{W}^{*}\left( \mathbf{V} \right) \right)'\left( \mathbf{Y}_{Greece}-\mathbf{Y}_{0}\mathbf{W}^{*}\left( \mathbf{V} \right) \right)$ (4)

where $\mathbf{Y}_{Greece}$ denotes pre-austerity infant mortality rate of Greece and $\mathbf{Y}_{0}$ captures a variety of linear combinations of pre-austerity mortality rate of the unaffected countries that represent potential synthetic control units. To ensure that optimal non-negative weights exist as a convex linear combination of control countries, it is necessary that the interpolation biases are not present (Abadie 2021). Under the regularity of these conditions, the average treatment effect of austerity policy on infant mortality can be written as:

$\hat{\theta}_{1}=\frac{1}{\left( T-T_{0} \right)}\cdot\sum_{t>T_{0}} \left( Y_{1t}-\sum_{j=2}^{J+1} w_{j}^{*}Y_{j,t}^{N} \right)$ (5)

Furthermore, the synthetic representation of Greece in the pre-austerity period reinforces the estimated unobserved counterfactual from the latent factor provided that the set of weights is identified in the inner and outer optimization which implies that the outcome based on reweighting the synthetic control group is as follows:

$Y_{W,t}=\sum_{j=2}^{J+1} w_{j}\cdot Y_{j,t}=\eta_{t}+\pi_{t}\cdot\left( \sum_{j=2}^{J+1} w_{j}Z_{j} \right)+\mu_{t}\cdot\left( \sum_{j=2}^{J+1} w_{j}\phi_{j} \right)+\left( \sum_{j=2}^{J+1} w_{j}\varepsilon_{j,t} \right)$ (6)

provided that pre-austerity period is sufficiently large and that interpolation bias issues are properly mitigated, the synthetic control estimator provides a plausible representation of the counterfactual scenario in response to the austerity under time-varying heterogeneity. It should be noted that we divide the pre-intervention period into the training and validation period to choose $\boldsymbol{V}$ such that the resulting synthetic control unit can plausibly approximate the outcome trajectory before the austerity policy. Provided that the number of pre-austerity periods is large enough, for a given $\boldsymbol{V}$, $\boldsymbol{W}$ can be computed directly using the covariate matrix from the training period to minimize the mean squared prediction error during the validation period when the set of weights is produced.

*S2.2 Inference*

Since large-sample asymptotic inference is not possible with synthetic control method, Abadie et. al. (2010) advocate the use of treatment permutation method for inference on the treatment effect of interest (Abadie, Diamond, and Hainmueller 2010). In our setup, we undertake a simple treatment permutation in space to detect whether the effect of the austerity measures on infant mortality is statistically significant at conventional levels. Treatment permutation consists of the iterative assignment of the austerity shock to the unaffected countries and use the outcome gaps to build the appropriate distribution of placebo effect. We proceed in two steps. In the first step, we estimate the placebo treatment effect by assigning the 2009 austerity package to each unaffected country in the OECD donor pool. In the second step, we compute the fraction of countries having the placebo effect at least as large as that of Greece. Our intuition is simple and straightforward. If the effect of austerity is both negative and statistically significant, the outcome gap should be unique to Greece and not perceivable elsewhere in the donor pool. By contrast, if the effect of austerity is not statistically significant, the placebo distribution should indicate no difference in the estimated effect of austerity for Greece and the placebo runs. Therefore, in step two, we calculate the empirical p-value for effect of austerity package on infant mortality in Greece from a simple two-tailed empirical distribution:

$$\mathbb{p}_{i,t}=\frac{\sum_{j=2}^{J+1} \boldsymbol{1}\cdot\left\{ \hat{\theta}_{j\in J+1}\geq\hat{\theta}_{1} \right\}}{J}$$

where $\mathbb{p}_{i,t}$ denotes two-tailed empirical p-value for the estimated dynamic treatment effect of austerity, $\hat{\theta}_{j\in J+1}$ denotes the full-sample placebo effect, $\hat{\theta}_{1}$ designates the estimated treatment effect of war on Greece, and $J$ indicates the size of the donor pool. Notice that the empirical p-value represents the probability to obtain dynamic average treatment effect at least as large as the one for Greece. However, if the prediction accuracy in the placebo simulation is low, the null hypothesis on the treatment effect of civil war is prone to over-rejection given a relatively large rarity of obtaining a large placebo effect. To address the type-II error, we compute pre-austerity average prediction error and constrain the inference procedure to ensure that p-values are estimated only if the prediction error in each placebo simulation is lower or equal to the mean prediction error for Greece. In addition, we discard the countries with mean prediction error at least four times that of Greece to partially eliminate the possibility of under-rejecting the null hypothesis that may arise if poorly fit placebos were included in the simulation. Our intuition behind the intertemporal behaviour of empirical p-values is two-fold. First, if the accuracy-adjusted p-values are consistently low from the early years of austerity onward, the analysis may highlight the austerity as a source of persistent and elevated increase in infant mortality. And second, if the adjusted p-values by the end of the sample are high, then the notion of austerity as a temporary negative shock with few long-term implications becomes more salient. Albeit imperfect by default, such inference procedure allows us to detect whether the austerity policy has led to a permanent deterioration on infant mortality trajectories to evaluate the significance of the effect in greater detail.
