# Supplementary material for: Effect of Austerity Measures on Infant Mortality: Evidence From Greece
Source: Health Econ. 2026 Apr 16;35(8):1175–91. doi: 10.1002/hec.70107 (PMC13327493; doi:10.1002/hec.70107)
Supplement: Supplementary file 3 — Supporting Information S3 [file HEC-35-1175-s003.docx]

**Supplementary Appendix 3: Additional robustness checks**

*S3.1 Differential trend analysis*

One of concerns arising from the estimated infant mortality effect of austerity measures hinges on the nature of the shock. Given that the infant mortality gap between Greece and each variant of its synthetic control group unfolds immediately and does not disappear up to the present day, the question that arises is whether the austerity measures induced a permanent and differential change in infant mortality rate. In the absence of a permanent differential change, the notion of long-lasting impacts of the austerity measures would be questionable as the absence of the break would have indicated a more transitory and temporary effect of austerity measures on the mortality rate. A decline of infant mortality both in Greece and its OECD-level synthetic control group after 2015 may posit abundant intuitive evidence in support of the temporary effect of austerity policies on infant mortality. Conversely, if our evidence corroborates the notion of a reasonably strong upward structural break in infant mortality trajectory that can be properly differentiated from the post-intervention trend in the synthetic control groups, the notion of the more permanent effect of austerity policy under such conditions becomes more credible. To assess whether the austerity policies proliferated a permanent or temporary increase in infant mortality we apply the differential trend assumption test. This approach as been applied to test the differential effect of the trans-fats ban on cardiovascular mortality rate in Denmark (Spruk and Kovac 2020), and further extended into a variety of applications ranging from the human development effects of civil wars (Kešeljević and Spruk 2024) and long-term effects of oil discoveries (Gilchrist et al. 2023).

Testing the differential trend assumption in the single-treatment setup of synthetic control estimator is both simple and intuitive. If the austerity measures triggered a temporary and weak infant mortality effect, the mortality gap between Greece and synthetic control group in the post-intervention period relative to the period before the intervention should be negligible and statistically insignificant at conventional levels. Conversely, if the austerity polic produced a permanent and reasonably strong break in the trend of mortality, the respective mortality gap between Greece and its synthetic counterparts should differ both markedly and statistically significantly in the post-austerity period whilst zero gap should be detectable in the pre-austerity period if the quality of the fit in the designated period is good. The null hypothesis behind the differential trend assumption can be tested by computing the supremum test statistics on the triple-difference interaction coefficient in the semi-difference-in-differences regression of mortality rate gap on the indicator of Greece and post-austerity period in the presence of both observed and unobservable confounders. To address the potential endogeneity between the austerity measures and infant mortality rate, we include two lags of the mortality gap in each semi-DiD regression specification to partially rule out the reverse causation channel from the inference.

Table S1 reports the differential trend assumption test on the change in the gap of infant mortality between Greece and its synthetic control group in the post-austerity period relative to the pre-austerity benchmark period. Under the null hypothesis, the mortality gap between Greece and its synthetic control group exhibits no structural break and is not characterized by statistically significant and perceptible structural break in the post-austerity period. The evidence suggests that the null hypothesis of no differential structural break in mortality gap can be rejected at 1% significance threshold. Using either full mortality specification or the gender-disaggregated ones, we find evidence of strong and statistically significant upward break in the mortality trajectory of Greece relative to its synthetic control group (i.e. p-value = 0.000). This supports the finding that the austerity policies did not lead to a mere temporary increase in infant mortality but instead promulgated a long-term effect. . Figure S1 depicts post- vs. pre-austerity trends in the mortality gap between Greece and its synthetic control group alongside the 95% confidence intervals.

**Table S1**: Testing differential trend assumption behind infant mortality trajectories

|  | Full distribution | Girls | Boys |
| --- | --- | --- | --- |
| $\chi^{2}$ test statistics  (p-value) | 31.17  (0.000) | 45.08  (0.000) | 43.81  (0.000) |
| Exogenous variable #1 | ${\Delta Y}_{j=Greece,t-1}$ | ${\Delta Y}_{j=Greece,t-1}$ | ${\Delta Y}_{j=Greece,t-1}$ |
| Exogenous variable #2 | ${\Delta Y}_{j=Greece,t-2}$ | ${\Delta Y}_{j=Greece,t-2}$ | ${\Delta Y}_{j=Greece,t-2}$ |
| Notes: the table reports Spruk and Kovac (2020) differential trend assumption test. Under the null hypothesis, the austerity policy does not induce a differential trend of the mortality difference between Greece and its synthetic control group, and both exhibit similar differenced mortality trend between pre- and post-austerity period. The table reports the p-values on the exact Chi-square test statistics of the Chow (1960) structural break in the triple-differences structural setup of the model. Two-sided p-values on the test statistics are reported in the parentheses. | | | |

**Figure S1**: Differential pre- and post-austerity trends in infant mortality gaps, 1991-2020

*S3.2 Leave-one-out analysis and sparsity conditions*

Another internal validity concern of the estimated mortality effect is the composition of the synthetic control groups that best reproduce both the overall and gender-specific mortality trajectories prior t austerity. In the original and most parsimonious specification, the Greek mortality trajectory prior to 2009 is best reproduced by the weighted combination of convex attributes of Iceland, Estonia, Portugal and several others with minor weight shares. Among the donors with non-zero weight, Iceland represents almost one half of the synthetic control group. Around the same time as Greece, Iceland was severely hit by the economic and financial crisis with an estimated GDP decline by 7.6 percent in 2008 and 2.8 percent in 2009^[[1]](#footnote-1)^ which implies that SUTVA assumption of stable treatment value assignment may be partially violated. To address this particular concern, we re-estimate the treatment effect of austerity measures on infant mortality rates by performing leave-one-out analysis (Abadie, Diamond, and Hainmueller 2015; Klößner et al. 2018) and exclude Iceland as the major donor from the pool of comparison as it may posit potentially excessive and high-leverage influence on the magnitude and significance of the treatment effect of austerity measures.

Figure S2 presents leave-one-out re-evaluation of the treatment effect of austerity measures without Iceland in the donor pool. Overall, Greek infant mortality trajectory prior to the austerity policy is best reproduced through a convex combination of implicit attributes of Luxembourg (61 percent), Ireland (17 percent), Latvia (13 percent), and Estonia (9 percent), respectively. A slightly more nuanced but very similar composition of the synthetic control group is inherent in gender-disaggregated estimates of synthetic mortality curves. By and large, leave-one-out estimates of the mortality effect of austerity measures confirm our baseline estimates. More specifically, the average treatment effect of austerity policies on infant mortality is around 717 infant deaths in each year of austerity (p-value = 0.000). The cumulative infant mortality toll is around 7,891 infant deaths more than implied by the synthetic control group between 2010 and 2020 (i.e. end-of-sample p-value = 0.000). The average treatment effect of austerity on infant mortality of boys is around 27 percent higher than for girls. In a similar vein, the cumulative mortality toll for boys is 30 percent higher than for girls and the gender-specific difference is statistically significant at 1 percent (i.e. p-value = 0.000), and should be noted that the comparison of cumulative toll invariably implies that the boy death toll surpassed 10,000 while the girl death toll is around 7,891 in comparison with the synthetic control group. To tackle the aggregate uncertainty of the estimates, we invert the post-austerity test statistics through sparsity matrix of non-zero donors and compute the empirical 95% confidence intervals for each post-treatment year and all three mortality trajectories. Despite some uncertainty, it becomes apparent that post-austerity mortality gaps are both large and statistically significant, and confirm long-lasting mortality spike which appears somewhat stronger for boys. In spite of the shrinking mortality gap after 2015, large and statistically significant difference in mortality rates between Greece and its synthetic peers is prevalent up to the end-of-sample period. Figure S3 reports gender-disaggregated average infant mortality change alongside 95 percent confidence intervals obtained through cross-validation in leave-one-out analysis.

**Figure S2**: Leave-one-out analysis of the infant mortality effect of austerity measures, 1990-2020

**Figure S3**: Effect of austerity measures on infant mortality under sparsity conditions, 1991-2020

*S3.3 Varying the composition of the donor pool*

A related but somewhat distinctive salient objection to the internal validity of our estimates may arise from the composition of the donor. A chief concern is posited by the comparison of Greece to an overly diverse donor pool of OECD countries where widely different trajectories and rates of infant mortality are perceptible in the range between 1.7 deaths per 1,000 live births in Estonia (in 2020) and 52 deaths per 1,000 live births in Türkiye in 1991. As pointed out by Abadie (2021), an overly diverse and large donor pool may increase the risk of over-fitting since a large size of the donor pool immediately translates into a larger number of discrepancies in factor loadings, biasing the synthetic control estimates. To expedite a judicious decision on the size of the donor pool based on the similarity of observed values of mortality within a common geographic space, we shrink the composition of the donor pool to the sample of Mediterranean states which belong to EUMED7 group within the European Union^[[2]](#footnote-2)^ and Türkiye, which yields a substantially less diverse and more compact donor pool. Using a coarsened donor pool of Mediterranean states, we estimate the infant mortality effect of austerity measures using the classical synthetic control estimator and full-outcome path optimization.

Table S2 reports the overall and gender-disaggregated infant mortality effect of austerity measures using a reduced donor pool consisting of the Mediterranean states. The implicit attributes of the Mediterranean states in Europe provide a very good quality of fit with the pre-austerity infant mortality trajectories of Greece. In particular, the overall infant mortality trajectory of Greece in the pre-austerity period is best reproduced by the convex combination of the mortality rates of Cyprus (64 percent), Spain (30 percent), and Malta (6 percent), respectively. One half of the girls’ mortality trajectory in the pre-austerity period can also be synthesized by Cyprus, followed by Italy (40 percent), Croatia (9 percent), and Malta (<1 percent). In a similar vein, the synthetic version of Greece in terms of boys’ infant mortality dynamics prior to the austerity measures consists of Cyprus (43 percent), Slovenia (30 percent), Malta (10 percent), and Croatia (6 percent).

**Table S2**: Infant mortality effect of austerity measures in Greece using Mediterranean donor pool, 1990-2020

|  | Overall | Girls | Boys |
| --- | --- | --- | --- |
| Average mortality gap (p-value) | +0.857  (0.000) | +0.733  (0.000) | +0.878  (0.000) |
| End-of-sample mortality gap (p-value) | +0.825  (0.000) | +0.818  (0.000) | +0.938  (0.000) |
| RMSE | 0.229 | 0.172 | 0.291 |
| R2 | 0.98 | 0.98 | 0.97 |
| # control units | 10 | 10 | 10 |
| Bias | 0.15% | <0.1% | 0.15% |
| # pre-austerity outcomes | 19 | 19 | 19 |
| Composition of synthetic control groups |  |  |  |
| Croatia | 0 | 0.09 | 0.06 |
| Cyprus | 0.64 | 0.50 | 0.43 |
| France | 0 | 0 | 0 |
| Israel | 0 | 0 | 0 |
| Italy | 0 | 0.40 | 0 |
| Malta | 0.06 | <0.01 | 0.10 |
| Portugal | 0 | 0 | 0 |
| Slovenia | 0 | 0 | 0.30 |
| Spain | 0.30 | 0 | 0.11 |
| Türkiye | 0 | 0 | 0 |

The estimates show that the average mortality effect of the austerity measures in the post-intervention period is around +0.857 additional deaths per 1,000 live births (p-value = 0.000) whereas the boys mortality effect appears to be around 19 percent higher than the girls’ mortality effect. By the end-of-sample period, the disparity in the gender-specific mortality effect lingers at 14 percent, and is statistically significant (p-value = 0.000) which confirms our prior findings. It should also be noted that the synthetic counterfactuals constructed from the Mediterranean donor pool of states yields the mortality gaps similar to our baseline of +0.85 basis point increase for the overall mortality, +0.69 basis point increase in girls’ mortality, and +0.88 basis point increase in boys’ mortality. Figure S4 reports the corresponding infant mortality gaps based on the Mediterranean donor pool of states.

**Figure S4**: Effect of austerity measures on infant mortality using the donor pool of European Mediterranean countries, 1991-2020

*S3.4 Generalized synthetic control estimates*

The empirical analysis uncovers the evidence of substantial increase in infant mortality in response to the austerity policies. One of the main advantages of the synthetic control method lies in the easing of the parallel trend assumption between Greece and the donor pool of OECD countries given the uniqueness and severity of troika-imposed austerity measures. Yet, our model specification does not incorporate unobservable component into the projection and estimation of the counterfactual scenario whilst several challenges may arise when models with unobserved heterogeneity are estimated to evaluate the effect of austerity policy or related intervention of interest. To fill the void in the literature, Xu (2017) proposed a generalized version of the synthetic control method that unifies the counterfactual estimation based on the interaction of country-specific and year-specific effects to estimate the counterfactual (Xu 2017). Under this particular setup, the treatment effect is estimated using semi-parametric approach where the counterfactual for the treated unit is based on the linearly interactive fixed-effects model with time-varying coefficients interact with unit-specific intercepts. The respective counterfactual outcome trajectory is imputed from the observed outcome trajectory through a cross-validation that automatically selects the model with the best fit instead of arbitrary imposing covariates or pre-treatment outcomes. In turn, the risk of over-fitting is reduced the possibility of using the specification where many seemingly irrelevant auxiliary covariates are incorporated is mitigated inasmuch as possible. Compared to the classical synthetic control estimator, its generalized version uses the data from the pre-intervention period as a benchmark to derive the reweighing scheme for the potential control units and yields the best possible prediction of the counterfactual scenario.^[[3]](#footnote-3)^

Table S3 reports interactive fixed-effects synthetic control estimates of the infant mortality effect of austerity measures for the period 1990-2020. More specifically, the table shows the average mortality differential relative to the synthetic counterfactual projected from the interactive of country- and time-specific effects in the data. The results confirm a large-scale and statistically significant increase in the mortality rate. Estimates reported in column (1) suggest that in the post-austerity period, overall infant mortality differential between Greece and its synthetic counterfactual is around 55 percent (p-value = 0.000). The estimated girls mortality differential is around 23 percent lower compared to boys whilst both differentials are statistically significant at 1% level. We tackle the relative uniqueness of the estimated mortality differentials by using the combined placebo test, performing both in-space permutation of the austerity policies to the unaffected countries as well as the assignment of the policy intervention to the deliberately wrong year. The evidence suggests that the increase in Greek infant mortality rates is unique and the placebo distribution of falsely assigned intervention does not appear to be similar to Greece, as we fail to reject the null hypothesis consistently. Furthermore, Figure S5 presents the estimated average treatment effect of austerity policy on infant mortality rate of Greece across the full post-intervention period. It also reports the in-time placebo analysis where the placebo year is selected through a cross-validation procedure indicating the year in which the post-intervention mortality increase may be most likely triggered by the year-specific events and policies. The evidence invariably suggests that the null hypothesis of in-time placebo effect cannot be rejected at conventional significance thresholds.

**Table S3**: Interactive fixed-effects synthetic control estimated infant mortality effect of austerity measures, 1990-2020

|  | Overall | Girls | Boys |
| --- | --- | --- | --- |
|  | (1) | (2) | (3) |
| A.T.T. | +1.552  (.354) | +1.338  (.343) | +1.725  (.361) |
| Simulation-based p-value | 0.000 | 0.000 | 0.000 |
| Two-tailed 95% confidence interval | (1.269, 2.585) | (1.205, 2.325) | (1.384, 2.795) |
| Combined placebo test  (p-value) | YES  (0.723) | YES  (0.702) | YES  (0.472) |
| Notes: the table reports the average treatment effect (ATT) of the austerity measures in Greece on infant mortality for the period 1990-2020 using interactive fixed-effects algorithm of the generalized synthetic control estimator. Standard errors are adjusted for serially correlated stochastic disturbances using cluster-robust error component model and finite empirical distribution function, and are reported in the parentheses. | | | |

**Figure S5**: Full placebo-region analysis of the infant mortality effect of austerity measures in Greece, 1990-2020

*S2.5 Synthetic difference-in-differences estimates*

The comparison of mortality trajectories implies that Greece followed a parallel trend but yet experience a unique imposition of deep cuts in public health care spending. Thus, parallel trend assumption can be questioned. Instead of deliberately selecting the synthetic control estimator where parallel trend assumption is relaxed or difference-in-differences estimator where the assumption should hold, Arkhangelsky et. al. (2021) proposed synthetic difference-in-differences estimator leveraging a similar control group that approximates the outcome dynamics of the treatment group the absence of the treatment (Arkhangelsky et al. 2021). The proposed estimator generates an optimally matched control group that loosens the necessity of parallel trend assumption, and relaxes both parallel trend assumption and the convex hull requirement to seek common support for the treated unit’s characteristics based on the attributes within the convex hull. One of the notable advantages of the synthetic difference-in-differences estimator lies in its invariance to the additive shifts in the weight structure which poses a substantial improvement over synthetic control estimator. By balancing pre-intervention trends not only through country-level weights but also with time-varying weights, two-way fixed effects localized estimator can estimate the appropriate average treatment effect consistently.

Table S4 reports the magnitude of the overall and gender-specific infant mortality increase in Greece in response to the introduction of troika-imposed austerity policies in the period 2010-2020 based on the counterfactual imputed from country- and year-level weights. Specifically, the table reports the magnitude of the average treatment effect alongside two-tailed 95% confidence bounds and large-sample approximated empirical p-value on the null hypothesis. The evidence based on the synthetic difference-in-differences estimates confirms a sizeable increase in infant mortality in response to austerity measures by around 1.12 deaths per 1,000 live births relative to the estimated counterfactual (p-value = 0.003). Consistent with the earlier findings, the estimated effect is 14 percent higher for boys compared to girls. The evidence reaffirms the earlier results and indicates that the mortality increase in response to the austerity measures is both substantial and long-lasting. Figure S6 depicts synthetic difference-in-differences infant mortality effect along with the outcome diagnostic plot.

**Table S4**: Synthetic difference-in-differences estimated effect of austerity measures on infant mortality in Greece, 1991-2020

|  | Overall | Girls | Boys |
| --- | --- | --- | --- |
|  | (1) | (2) | (3) |
| $\gamma={IMR}_{Greece,t>T_{0}}-{IMR}_{Synthetic,t>T_{0}}$. | +1.128  (0.375) | +1.032  (0.338) | +1.186  (0.669) |
| Large-sample approximated p-value | 0.003 | 0.002 | 0.076 |
| Two-tailed 95% confidence interval | (0.393, 1.863) | (0.368, 1.694) | (-0.125, 2.497) |
| Notes: the table reports synthetic difference-in-differences estimated effect of the austerity measures on overall and gender-specific infant mortality rate (IMR) by matching Greece’s pre-austerity infant mortality trajectories with a donor pool of 38 members of the Organization for Economic Cooperation and Development (OECD). The table reports the weighted difference between each designated Greek infant mortality trajectory and its control group based on the localized two-way fixed effect estimator using outcome model includes latent country-level factors interacted with latent time factors. It also reports large sample-approximated empirical p-value on the null hypothesis behind the average treatment effect. The lower and upper bound of the two-tailed 95% confidence interval and the standard error of the average treatment effect are reported in the parentheses. | | | |

**Figure S6**: Synthetic difference-in-differences adjusted infant mortality effect of austerity measures in Greece, 1991-2020

*S2.6 LASSO synthetic control estimates*

Thus far, our empirical analysis rests on the intrinsic assumption of common support. This implies that the synthetic versions of Greece used to track, reproduce and synthesize Greek infant mortality trajectories consist of the weighted combination of OECD or Mediterranean countries attributes that belong to the convex hull of Greece’s mortality trajectory based on the weights obtained in the training and validation stage. The common support assumption fundamentally allows for the extrapolation inside the convex hull of Greece and may be overly restrictive (Ben-Michael, Feller, and Rothstein 2021). To fill the void in the literature, Hollingsworth and Wing (2020) relax the convexity assumption and derive a more flexible version and regularized version of synthetic control estimator relying on LASSO-based machine learning algorithm that permits the extrapolation outside the convex hull (Hollingsworth and Wing 2020). This implies that both positive and negative weights for the treated unit’s counterfactual can be estimated. The former indicate similarity with the treated unit whereas the latter designate some degree of dissimilarity. Such countercyclical weights without the additive restriction to unity facilitate improved flexibility and more nuanced estimation of the counterfactual with substantially reduced pre-treatment imbalance. If the exposure of treatment under potential outcomes satisfies conditional independence assumption and no major structural break is perceptible in the pre-intervention period, the treatment effect behind the austerity measures can be plausibly identified using a latent factor model.

Figure S7 shows the LASSO synthetic control estimated effect of austerity measures on overall and gender-specific infant mortality rate using two distinctive variants of the donor, namely, the coarsened donor pool of OECD countries and a more compact and salient donor pool of Mediterranean states. A closer inspect of the pre-austerity mortality gaps self-suggests that it becomes apparent that the LASSO-supported synthetic control estimator provides an excellent fit of the mortality trajectories between Greece and its synthetic counterparts indicated both visually as well as judged on the basis on RMSE which is consistently below 1 percent of the pre-treatment error margin. The evidence confirms the overall infant mortality increase of around 1.16 additional deaths per 1,000 live births (p-value = 0.000). Under countercyclical weights, the estimated effect using the full OECD donor pool is comparable between boys and girls. The overall infant mortality dynamics of Greece prior to the austerity measures is best synthesized by the convex combination of attributes of Malta (26 percent), Ireland (21 percent), and Latvia (20 percent) alongside a handful of other OECD donors with smaller non-zero weight. By shrinking the donor pool to the Mediterranean states to capture more salient features of pre-austerity infant mortality of Greece, the synthetic control group loads positively on Malta (+42 percent), Cyprus (+25 percent), Türkiye (+15 percent), Portugal (+14 percent), Croatia (+2 percent), and Slovenia (<1 percent), and negatively on Italy (-10 percent), France (-51 percent) and Spain (-52 percent). The estimated average increase in infant mortality implied from the comparison with other Mediterranean states is around 1.04 additional deaths per 1,000 live birth, respectively (i.e. p-value = 0.000). Aligned with our prior estimates, the magnitude of the average mortality gap is approximately 20 percent higher for boys compared to girls, and reiterates additional support for our baseline findings. Figure S8 reports the composition of overall and gender-disaggregated synthetic control groups under countercyclical weights in greater detail.

**Figure S7**: LASSO synthetic control estimated infant mortality effect of austerity measures in Greece, 1991-2020

**Figure S8**: Composition of synthetic control groups across LASSO-based synthetic control estimation

By way of example, the composition of the synthetic versions of Greece is similar to thatobtained in the baseline analysis of the infant mortality effect of austerity measures. In the overall mortality specification, the convexity requirement for ascribe common support for pre-austerity mortality dynamics is met. The synthetic version of Greece that best reproduces its pre-austerity mortality trajectory consists of Malta (26 percent), Ireland (22 percent), Latvia (20 percent), Croatia (16 percent), Chile (8 percent), Costa Rica (7 percent), and New Zealand (3 percent). Shrinking the OECD donor pool to the European sample of Mediterranean countries yields very similar composition of the synthetic version of Greece. In the overall mortality LASSO estimator with countercyclical weights, Greece’s synthetic control group loads positively of Malta (42 percent), Cyprus (25 percent), Türkiye (15 percent), Portugal (14 percent), Croatia (2 percent) and negatively on Italy (-10 percent), France (-51 percent), and Spain (-53 percent) while Slovenia receives zero weight.

*S3.7 Effects of austerity policies on neonatal and post-neonatal mortality*

One the remaining questions behind the estimated effect of austerity-imposed reduction in public health spending on infant mortality hinges on the broader generalization of the effect. To gauge the generalizability of the estimated effect, the outcome variable can be expanded to determine whether higher rates of mortality can be detected in response to austerity policies beyond the infancy stage. To address this question, we expand our analysis in two steps. First, we exploit the differences in neonatal mortality rate across OECD countries and use the synthetic control estimator to project the counterfactual scenario of neonatal mortality in the hypothetical absence of the austerity measures. Since the first month of life is considered to be the most vulnerable period for child survival and nearly one half of all deaths in children under the age of 5 occur in the first 28 days of life, neonatal mortality rate is an equally relevant indicator to considered in the evaluation of the effect of austerity policies on mortality rate. Premature birth, birth complications such as asphyxia and trauma as well as neonatal infections and congenital anomalies have been cited by World Health Organization as the leading causes of death in the neonatal stage. The question that arises immediately is whether austerity measures designed to curb the expenditure on public health care have affected neonatal mortality to the similar degree as infant mortality. And second, we also exploit cross-country differences in post-neonatal and under-five mortality rate and examine the contribution of austerity measures to the rate of mortality in the post-neonatal stage. By applying the synthetic control estimator to Greece and using the full OECD donor pool to ascribe the implicit attributes of mortality in the pre-austerity period, we estimate the respective counterfactual scenario.

Figure S9 illustrates the estimated neonatal mortality effect of the austerity measures. The point estimates of the mortality gap in the post-intervention period indicate a marked and permanently upward derailed mortality trajectory after the implementation of austerity measures. The estimated average increase in the mortality rate is around 0.798 (p-value) which is the equivalent of around 800 estimated live births ending with the death in the first 28 days of birth per each year of the austerity policies. The effect appears to be immediate and tends to increase gradually up until 2017 whilst remaining constant up to the end-of-sample post-treatment year. The synthetic version of Greece that best reproduces the neonatal mortality dynamics prior to the austerity package is consistent with our baseline composition, and consists of Iceland (48 percent), Estonia (33 percent), Ireland (13 percent), Latvia (5 percent), and Mexico (1 percent). The estimated increase in neonatal mortality is apparently permanent as the null hypothesis on the treatment effect of austerity policies can be very easily rejected for each post-intervention period. We also find evidence of elevated childhood mortality in the post-neonatal period up to the fifth year of life. By indicating the probability of death before the age of five under age-specific mortality rate, under-five mortality rate can be considered a baseline indicator of progress towards assuring children’s rights to life, health care, nutrition, water, social security and protection. Our estimates indicate a severe deterioration of under-five mortality rate after the imposition of austerity measures. Pointwise, we find the average treatment effect of austerity around 0.788 (p-value = 0.000) which is very close to the baseline and neonatal estimate, and also is statistically significant at the conventional levels which indicates that around 92 percent of the deaths (=0.797/0.851) were concentrated in the infancy or neonatal stage, and the remainder in the post-neonatal stage. Furthermore, Figure S10 presents gender-disaggregated neonatal and post-neonatal mortality gaps, and shows that the in the post-neonatal stage, the average mortality increase in response to austerity is around 30 percent (=0.913/0.707) higher for boys compared to girls.

**Figure S9**: Post-infancy childhood mortality effect of austerity measures in Greece, 1991-2020

**Figure S10**: Gender-disaggregated effect of austerity policy on post-neonatal mortality in Greece, 1991-2020

1. World Economic Outlook Database: April 2024

   <https://www.imf.org/en/Publications/WEO/weo-database/2024/April> [↑](#footnote-ref-1)
2. Croatia, Cyprus, France, Italy, Malta, Portugal, Slovenia and Spain [↑](#footnote-ref-2)
3. In terms of further detail, the generalization of the classical synthetic control estimator proceeds in two distinctive steps. First, it allows for many treated units under the differential timing of the intervention. And second, the generalization also provides some estimate of uncertainty since standard errors and confidence bounds may be derived analytically and estimated empirically which eases the interpretation of the estimates considerably, further improving inference. [↑](#footnote-ref-3)
