# Supplementary material for: Effect of Austerity Measures on Infant Mortality: Evidence From Greece
Source: Health Econ. 2026 Apr 16;35(8):1175–91. doi: 10.1002/hec.70107 (PMC13327493; doi:10.1002/hec.70107)
Supplement: Supplementary file 4 — Supporting Information S4 [file HEC-35-1175-s004.docx]

**Supplementary Appendix 4: Selection into birth and austerity measures**

*S.4.1 Baseline estimates*

One of the caveats behind the estimated infant mortality effect of austerity measures hinges on the changes in the total number of births which raises the concern that selection into birth could have changed in response to the austerity measures. To address this issue, we employ a synthetic control estimator that captures and reproduces Greece’s pre‐austerity birth dynamics by constructing a weighted combination of OECD countries whose birth trajectories fall within the convex hull of Greece’s observed dynamics, and estimate the counterfactual trajectory of birth dynamics. In the pre‐intervention period, the synthetic control model accounts for approximately 97 percent of the overall variance in Greece’s birth counts, indicating that the fitted trajectory closely tracks the actual births before austerity. The mean pre‐austerity discrepancy between Greece and its synthetic counterpart is less than 0.5 percent, reflecting a high‐quality match and reducing the likelihood that pre‐existing differences drive the post‐intervention results. Figure S4.1 displays the estimated impact of austerity on births, the evolution of treatment weights over time, the composition of the synthetic control group, and bias assessments for the matching period.

Figure S4.1. Effect of austerity measures on the number of births in Greece, 1991-2020

Following the onset of austerity, we observe a substantial decline in the number of births relative to the synthetic counterfactual. In the first year of austerity, births in Greece fell by approximately 3,612 compared with the synthetic control or roughly 5.7 percent relative to the pre‐austerity benchmark. The negative effect intensifies over time, reaching 23 percent by mid‐post‐intervention (2016) and peaking at a 28 percent decline in 2018. By the end of the sample period, the gap remains pronounced, with births about 24 percent lower than the pre‐austerity benchmark. Averaging over the entire post‐intervention window, the treatment effect corresponds to 23,182 fewer births than predicted by the synthetic control, or roughly a 20 percent reduction relative to the pre‐austerity level.

The synthetic control that best reproduces Greek birth patterns prior to austerity is composed predominantly of Israel (28 percent), Denmark (15 percent), Slovenia (15 percent), Ireland (14 percent), and Hungary (12 percent), with remaining donor countries each contributing less than 10 percent to the weighted combination. This mix of countries collectively mirrors Greece’s pre‐intervention demographic trends, lending credibility to the counterfactual scenario against which the post‐austerity birth declines are measured.

In Figure S4.2, we present a standard in-space placebo test by reassigning the austerity “treatment” to each of the donor‐pool countries in turn, thereby assessing whether any potential donor country exhibits a birth‐decline trajectory similar to Greece’s post‐austerity trend. The results from placebo analysis reveal that no other country experiences a quasi‐treatment effect comparable to Greece throughout the intervention period. Specifically, the share of donor‐pool countries whose post‐intervention birth declines mirror Greece’s is zero, and when comparing the ratios of post‐ to pre‐austerity root‐mean‐squared errors (RMSE), Greece’s RMSE ratio exceeds 1,800 or more than four times higher than Finland’s (the second‐ranked placebo) and over 1,000 times greater than those of all other countries. Thus, the placebo analysis confirms that the observed decline in births is unique to Greece and is not an artifact of time‐series noise or broader trends perceptible in OECD and Union for the Mediterranean countries.

Taken together, our findings indicate that austerity measures substantially altered selection into birth in Greece, leading to a pronounced reduction in the number of births relative to the synthetic control. The austerity measures, in turn, appear to have contributed to an elevated infant mortality rate when compared with the counterfactual trajectory. In other words, by depressing fertility among likely healthier and more resource‐endowed parents, or by delaying childbearing until economic conditions improved, the austerity policies amplified vulnerability among the infants who were born, resulting in higher mortality relative to what we would expect absent austerity.

Figure S4.2: Placebo analysis of the austerity measures effect on the birth dynamics in Greece, 1991-2020

To address concerns that changes in the total number of births may reflect compositional shifts in the childbearing population rather than an absolute fertility decline, we re‐estimate our analysis using the birth rate per 1,000 residents as the underlying outcome. By standardizing births by population, potential distortions arising from the fact that donor‐pool countries in the synthetic control mix vary considerably in population size are largely mitigated. In other words, raw birth counts could be overinflated in countries with larger populations or underrepresented in smaller ones, whereas the birth‐per‐1,000 measure ensures a more sanguine comparability across countries.

Using the birth rate, we find that Greece experienced an overall decline of approximately 11.6 percent relative to its pre‐austerity benchmark. The trajectory of this decline is gradual yet persistent. For instance, in the first year of austerity, the estimated drop in births per 1,000 individuals is roughly 7 percent, widening to about 14.6 percent by 2017. Although both the actual and synthetic versions of Greece show downward trends, the gap between them remains statistically and economically significant through the end of the sample period. The synthetic control for birth rates is constructed from countries whose demographic characteristics most closely match Greece’s pre‐intervention birth rate trajectory, specifically, the Netherlands (26 percent), Slovenia (24 percent), Israel (16 percent), and Australia (13 percent), with the remainder distributed among smaller contributing OECD member states. Importantly, none of these donor‐pool countries underwent comparable public health austerity measures during the study period, reducing concerns about violations of the stable‐unit treatment value assumption (SUTVA) or confounding from parallel shocks in the control group. Figure S4.3 presents these results in greater detail, including actual and synthetic birth rate trajectories, composition of synthetic control group, and bias assessments for the birth‐rate specification.

Figure S4.3: Effect of austerity measures on birth rate in Greece, 1991-2020

Lastly, In Figure S4.4, we present an in‐space placebo analysis by assigning the Greek austerity policy package to each donor‐pool country and re‐estimating the treatment effect for the birth rate per 1,000 residents. This procedure tests whether any donor exhibits a post‐“austerity” birth‐rate decline comparable to that observed in Greece. In turn, the results show that no other country’s birth rate trajectory mirrors Greece’s decline, as the proportion of donor countries with a quasi‐treatment effect similar to Greece remains effectively zero throughout the post-intervention period. Furthermore, when comparing the ratio of post‐ to pre‐austerity root‐mean‐squared errors (RMSE), Greece’s RMSE ratio exceeds 400 and is by more than one‐third higher than Switzerland (the second‐ranked placebo) and over 100 times greater than those of all other donor‐pool OECD member states. Taken together, the placebo findings confirm that the observed decline in Greece’s birth rate is uniquely attributable to its own austerity measures and is not an artifact of time‐series noise or broader OECD‐wide trends.

These results from the placebo analysis further reinforce our interpretation that austerity policies in Greece exacerbated adverse selection into birth, concentrating childbearing among potentially higher‐risk subpopulations. Given that healthier parents in higher socioeconomic classes may have delayed or foregone childbearing under financial strain, the remaining cohort of births was, on average, more vulnerable, driving up infant mortality after austerity took effect. In other words, by depressing birth decision among lower‐risk families, the policies amplified mortality risk among the infants who were born, producing a widening gap relative to the synthetic counterfactual.

Figure S4.4. Placebo analysis of the austerity measures effect on the birth rate in Greece, 1991-2020

*S.4.2 Translating mortality-rate effects into implied excess infant deaths*

The main analysis estimates the impact of the Greek austerity episode on infant mortality rates, defined as deaths before one year of age per 1,000 live births. For policy interpretation, it is often useful to translate estimated mortality-rate gaps into implied numbers of infant deaths. However, because infant mortality is a rate, such translations must be performed carefully. In particular, the large decline in births documented in this appendix implies that any conversion from rate differences to death counts must explicitly incorporate the realized number of births in each year. This sub-section therefore (i) states the conversion formula used, (ii) clarifies how the fertility decline affects interpretation, and (iii) reports the resulting excess-death calculations overall and by sex.

Let ${IMR}_{t\geq T_{0}}^{Greece}$ denote the observed infant mortality rate in Greece in year $t$, captured by the number of deaths per 1,000 live births, and let ${IMR}_{t\geq T_{0}}^{SC}$ be the corresponding synthetic counterfactual rate. Moreover, let $B_{t}$ be the observed number of live births in Greece in year $t$. The implied number of excess infant deaths in year $t\geq T_{0}$ attributable to the post-intervention divergence is computed as:

$$\Delta D_{t}=\left( {IMR}_{t\geq T_{0}}^{Greece}-{IMR}_{t\geq T_{0}}^{SC} \right)\times\frac{B_{t}}{1000}$$

The cumulative number of excess infant deaths over the post-austerity period $T$ is:

$$\Delta D=\sum_{t\in T} \left( {IMR}_{t\geq T_{0}}^{Greece}-{IMR}_{t\geq T_{0}}^{SC} \right)\times\frac{B_{t}}{1000}$$

Where we use observed birth in each year and therefore directly incorporate the fertility decline associated with the austerity. Importantly, the conversion avoids the common but inappropriate simplification of multiplying a rate gap by a fixed pre-intervention birth level. Because $IMR$ is expressed per 1,000 live births, a reduction in births affects the denominator. Mechanically, if the number of infant deaths were unchanged, a decline in births would raise the infant mortality rate even without any change in risk conditional on birth. This is why the fertility decline documented in Section S4.1 is not merely ancillary but directly relevant for interpreting both the rate estimates and any implied death counts. The conversion above addresses this concern transparently. When births fall, the same rate gap implies fewer absolute excess deaths than would be implied under a fixed-birth assumption. Conversely, if mortality risk rises while births fall, the observed mortality rate divergence may still imply a meaningful increase in deaths, but this must be quantified using realized births.

Applying the formula above to the estimated post-intervention mortality-rate gaps yields the implied number of excess infant deaths in each year of the post-austerity period. Using observed annual births, the cumulative excess infant deaths over 2010-2020 is approximately 854, corresponding to an average of about 78 excess infant deaths per year over the post-intervention window. To assess whether the burden differs by sex, we compute sex-specific excess deaths analogously. Let ${IMR}_{t\geq T_{0}}^{Greece,s}$ and ${IMR}_{t\geq T_{0}}^{SC,s}$ denote the observed and synthetic infant mortality rates for sex $s\in\left\{ g,b \right\}$ and $B_{t}^{s}$ be the observed number of live birth of sex $s$. Sex-specific excess infant deaths are computed as follows

$$\Delta D_{t}=\left( {IMR}_{t\geq T_{0}}^{Greece}-{IMR}_{t\geq T_{0}}^{SC} \right)\times\frac{B_{t}}{1000}, s\in\left\{ g,b \right\}$$

Using sex-specific births, the cumulative implied excess deaths over 2010-2020 is approximately 414 for girls and 451 for boys, indicating a systematically larger mortality burden among boys. Nonetheless, important clarification is ought to be stated. Sex-specific excess deaths need not sum exactly to the overall total. The overall, girls’, and boys’ effects are estimated using separate synthetic controls, with potentially different donor weights and counterfactual trajectories. Consequently, the overall rate gap is not mechanically constrained to equal a birth-weighted average of sex-specific rate gaps year-by-year, and additivity is not guaranteed. The key substantive comparison is therefore not an accounting identity, but the consistent finding that (i) the overall implied burden is sizable and persistent, and (ii) the sex-specific burden is larger for boys. For transparency, Table S4.1 reports the implied annual excess infant deaths computed from rate gaps and observed births.

**Table S4.1**: Implied excess infant deaths from post-austerity mortality-rate gaps, using observed births

|  | Treatment Effect | | | Observed Live Births (count) | | | Excess Deaths | | |
| --- | --- | --- | --- | --- | --- | --- | --- | --- | --- |
|  | Overall | Girls | Boys | Overall | Girls | Boys | Overall | Girls | Boys |
|  | (1) | (2) | (3) | (4) | (5) | (6) | (7) | (8) | (9) |
| 2010 | 0.21 | 3.2 | 3.01 | 114,766 | 55,639 | 59,127 | 24.10 | 10.57 | 12.42 |
| 2011 | 0.41 | 3.2 | 2.91 | 106,428 | 51,566 | 54,862 | 43.64 | 21.14 | 18.10 |
| 2012 | 0.49 | 3.2 | 2.81 | 100,371 | 48,717 | 51,654 | 49.18 | 23.87 | 26.86 |
| 2013 | 0.71 | 3.3 | 2.74 | 94,134 | 45,704 | 48,430 | 66.84 | 32.45 | 35.35 |
| 2014 | 0.99 | 3.4 | 2.67 | 92,149 | 44,765 | 47,384 | 91.23 | 44.32 | 48.33 |
| 2015 | 1.06 | 3.5 | 2.62 | 91,847 | 44,553 | 47,294 | 97.36 | 47.23 | 55.81 |
| 2016 | 1.21 | 3.5 | 2.56 | 92,898 | 45,016 | 47,882 | 112.41 | 54.47 | 58.89 |
| 2017 | 1.17 | 3.5 | 2.52 | 88,553 | 42,867 | 45,686 | 103.61 | 50.15 | 54.82 |
| 2018 | 1.14 | 3.4 | 2.45 | 86,440 | 41,915 | 44,525 | 98.54 | 47.78 | 52.09 |
| 2019 | 1 | 3.2 | 2.38 | 83,763 | 40,818 | 42,945 | 83.76 | 40.82 | 43.80 |
| 2020 | 0.97 | 3.1 | 2.32 | 84,764 | 41,233 | 43,531 | 82.22 | 40.00 | 44.84 |
| Cumulative |  |  |  |  |  |  | 852.88 | 412.80 | 451.33 |
